# The Spindle Assembly Checkpoint Functions during Early Development in Non-Chordate Embryos

**DOI:** 10.3390/cells9051087

**Published:** 2020-04-28

**Authors:** Janet Chenevert, Marianne Roca, Lydia Besnardeau, Antonella Ruggiero, Dalileh Nabi, Alex McDougall, Richard R. Copley, Elisabeth Christians, Stefania Castagnetti

**Affiliations:** Sorbonne Universités, CNRS, Laboratoire de Biologie du Développement de Villefranche-sur-mer (LBDV), 06234 Villefranche-sur-mer, France

**Keywords:** mitosis, spindle checkpoint, embryo, chordates

## Abstract

In eukaryotic cells, a spindle assembly checkpoint (SAC) ensures accurate chromosome segregation, by monitoring proper attachment of chromosomes to spindle microtubules and delaying mitotic progression if connections are erroneous or absent. The SAC is thought to be relaxed during early embryonic development. Here, we evaluate the checkpoint response to lack of kinetochore-spindle microtubule interactions in early embryos of diverse animal species. Our analysis shows that there are two classes of embryos, either proficient or deficient for SAC activation during cleavage. Sea urchins, mussels, and jellyfish embryos show a prolonged delay in mitotic progression in the absence of spindle microtubules from the first cleavage division, while ascidian and amphioxus embryos, like those of *Xenopus* and zebrafish, continue mitotic cycling without delay. SAC competence during early development shows no correlation with cell size, chromosome number, or kinetochore to cell volume ratio. We show that SAC proteins Mad1, Mad2, and Mps1 lack the ability to recognize unattached kinetochores in ascidian embryos, indicating that SAC signaling is not diluted but rather actively silenced during early chordate development.

## 1. Introduction

The mitotic checkpoint, also known as the spindle assembly checkpoint (SAC), operates during mitosis and monitors bipolar attachment of spindle microtubules to kinetochores, specialized multiprotein complexes assembled on duplicated sister chromatids. In the absence of stable kinetochore-microtubule attachments, SAC components are recruited to unattached kinetochores where they catalyze the generation of an inhibitory complex, called the mitotic checkpoint complex (MCC), which prevents activation of the anaphase-promoting complex/cyclosome (APC/C) and delays chromosome segregation and mitotic exit. When all chromosomes have achieved bipolar attachment to microtubules, the SAC is satisfied resulting in APC/C activation, which leads to the proteolytic cleavage of securin and cyclin B. Degradation of securin activates separase, thus resulting in cohesin cleavage and physical separation of sister chromatids, while cyclin B degradation inactivates Cyclin Dependent Kinase (CDK), resulting in mitotic exit [1,2]. This mechanism increases the fidelity of mitosis by preventing premature initiation of anaphase and subsequent generation of daughter cells with unequal chromosomal complements, a condition, known as aneuploidy, which is linked to cell and organismal lethality [3].

Despite the essential role of the SAC in achieving accurate chromosome segregation, genetic fidelity and reproductive success, this checkpoint is inefficient during early development of some animals. Microtubule perturbations that cause erroneous kinetochore-spindle associations do not trigger a robust spindle checkpoint response during the early rapid cell cycles (cleavage cycles) of embryonic development in fish and frog embryos. In *Xenopus laevis,* treatment with microtubule depolymerizing drugs does not delay the first 12 embryonic cycles and the associated oscillations of CDK activity, which continue with unchanged periodicity until the midblastula transition (MBT; [4,5]). Similarly, in zebrafish embryos, nocodazole treatment induces a metaphase arrest only after MBT [6,7]. In mice, which like all mammals has slow cleavage cycles compared to other animals, nocodazole treatment in 2-cell embryos causes a weak mitotic delay [8,9]. These studies framed the hypothesis that the SAC is weak or silenced in early animal embryos especially those that undergo fast cleavage divisions [4,7,10].

Contrary to this hypothesis, however, several earlier reports show that treatment with the microtubule depolymerizing drug colchicine delays cyclin B degradation and extends mitosis in embryos of the sea urchins *Arbacia punctulata* and *Lytechinus variegatus* [11,12] and the clam *Spisula solidissima* [13], and overexpression of MCC component Mad2 leads to a mitotic block in embryos of *Lytechinus pictus* [14]. Although these studies often predate SAC discovery and therefore the dependence of the mitotic delay on SAC activity was not directly tested, they suggest that the SAC may be effective in these embryos as early as the first embryonic cleavage. One explanation for this variability among species could be the dependency of SAC strength on cell size. This hypothesis was brought to the fore by a study on *Caenorhabditis elegans* embryos, which showed that the ratio of kinetochore number to cell volume influences the strength of SAC response [15]. Since a minimum signal threshold, dependent on the amount of Mad2 protein recruited on unattached kinetochores, needs to be reached to inhibit APC/C activity and elicit a SAC-mediated mitotic block [16], it was suggested that in large embryos, like those of fish and frogs, the SAC is functional but the signal generated by unattached kinetochores is too diluted to trigger a significant checkpoint response [15,17], whereas the SAC would be effective in smaller embryos like those of sea urchin and clam.

Here we use a comparative approach, combining both new experimental data and previous findings from the literature, to assess the variability in SAC response during the early cell cycles of embryonic development in species representative of the main metazoan groups. To complement the extensive data already available for vertebrates, we examined the mitotic response to complete microtubule depolymerization in early embryos of a range of invertebrate species. We found that lack of SAC activity is not a general feature of embryonic cleavage cycles. While ascidian (tunicate) and amphioxus (cephalochordate) early embryos, like previously studied fish and frog embryos (vertebrates), continue to cycle without spindles, sea urchin and starfish (echinoderm), mussel (mollusk), and jellyfish (cnidarian) embryos show a prolonged checkpoint-dependent mitotic block from the first division in response to spindle perturbations. This species specificity in SAC competence does not correlate with cell size, chromosome number, or kinetochore to cell volume ratio. Instead we show that recognition of unattached kinetochores by the SAC machinery is lost in SAC-deficient ascidian embryos, suggesting that lack of SAC activity during early development is not due to passive dilution of checkpoint signal in large cells, but instead the mitotic checkpoint is actively silenced in early embryos of many chordate species.

## 2. Materials and Methods

### 2.1. Gamete Collection and Fertilization

*P. lividus*, *A. lixula*, *S. granularis*, and *H. attenuata* adults were collected from the bay of Villefranche-sur-mer (France), *P. mammillata* and *M. galloprovincialis* at Sète (France), *C. intestinalis* at Roscoff (France), and *B. lanceolatum* at Argelès-sur-Mer (France). All these species were maintained in aquaria by Centre de Ressources Biologiques Marines (CRBM) at the Laboratoire de Biologie du Developpement de Villefranche-sur-mer (LBDV). *S. purpuratus* adults were obtained from Patrick Leahy (Kerchoff Marine Laboratory, California Institute of Technology, Pasadena, CA, USA) and kept in aquaria at University College London (UCL, London, UK).

*S. purpuratus* adults were induced to spawn by injection of 0.55 M KCl and all manipulations were carried out at 15 °C. For the other three sea urchin species, gametes were obtained by dissection and all manipulations were carried out at 18–20 °C; eggs were collected in microfiltered sea water (MFSW) and used within the day, whereas sperm was collected dry and maintained at 4 °C for up to a week. Prior to fertilization, eggs were filtered to remove ovarian tissue and debris (100 µm filter pore size for *P. lividus* and *S. granularis*, 70 µm for *A. lixula*). When removal of the fertilization membrane was required (for immunofluorescence), eggs were treated with 1× Fertilization Cocktail (10 µM 3-amino-1,2,4-triazole, 5 µM Ethylendiaminetetracetic Acid (EDTA), 200 µM Tris-HCl pH 8.2) for 2–3 min prior to fertilization to prevent hardening of the membrane. The fertilization membrane was removed by filtration (70 µm for *P. lividus* and *S. granularis* and 54 µm for *A. lixula*) and excess sperm was removed by rinsing twice in MFSW.

For *H. attenuata*, gametes were obtained by aspiration through a small hole in the starfish arm using a syringe with 18 G needle. Oocytes were immediately matured with 10 µM 1-methyladenine (1-MA, Sigma-Aldrich, St. Louis, MO, USA) and after 13 min they were fertilized in glass dishes and cultured at 21 °C.

For *P. mammillata* and *C. intestinalis*, gametes were obtained by dissection. Dry sperm was maintained at 4 °C, and eggs were dechorionated in 0.1% trypsin for *P. mammillata* or in pronase/thioglycolate for *C. intestinalis* as described [18]. All manipulations were performed at 18 °C in dishes coated with gelatin or agarose to prevent adhesion and lysis [19]. Prior to fertilization sperm was activated by resuspension in basic sea water (pH 9.2) for 20 min.

*B. lanceolatum* mature adults were maintained at 16–17 °C and induced to spawn by thermal shock at 23 °C for 36 h, as previously described [20]. Oocytes were collected in petri dishes and fertilized with a dilution of fresh sperm, and developing zygotes were incubated in MFSW at 19 °C [20].

*C. hemisphaerica* eggs and sperm were obtained by light-induced spawning from animals raised in the laboratory and maintained at 19 °C in artificial sea water [21].

*M. galloprovincialis* adults were maintained in sea water at 15 °C. To induce spawning, animals were placed in individual containers with sea water at 24 °C, after rigorous cleaning and brushing of animal shells. Oocytes were fertilized in petri dishes and incubated at 18 °C for development.

### 2.2. Drug Treatments

All drugs were maintained as stock solutions in DMSO at −20 °C and diluted as appropriate in MFSW prior to usage. Nocodazole (Sigma, 33 mM stock solution in DMSO) was used at a final concentration of 10 µM (unless otherwise stated), reversine (Axon Medchem, Groningen, The Netherlands, 5 mM stock solution in DMSO) was used at a final concentration of 0.5 µM, and AZ3146 (Santa Cruz Biotechnology, Dallas, TX, USA; stock solution 22 mM in DMSO) was used at a final concentration of 2.2 µM.

In all embryo experiments drugs were added when 90–95% of the embryos reached the 2-cell stage to avoid regression of the cleavage furrow, and drug treatment was then maintained for the entire duration of the experiment. Each experiment was repeated 3–5 times.

### 2.3. Generation of Mad1 and Mad2 Antibodies

To generate an antibody specific for ascidian Mad1, the *P. mammillata* Mad1 coding sequence fused to His-6 tag was expressed in bacteria from the T7 promoter using the pET11 plasmid. To render Mad1 soluble, the coding sequence was cut in two and separate N terminal (aa 1–408) and C terminal (aa 409–600) halves were produced. To generate the Mad2 antibody, the full length Mad2 coding sequence was fused to His-6 tag. The Mad1-His6 and Mad2-His6 fusion proteins were purified on nickel columns and used to immunize 4 mice for each protein (Covalab, Bron, France). All resulting Mad1 sera recognized a major band at 80 KDa in *P. mammillata* extracts and showed a similar labeling pattern in fixed material. Resulting sera for Mad2 recognized a major band at 23 KDa in *P. mammillata* extracts and a 27 KDa band when Mad2-tag was overexpressed by microinjection, however this did not work for immunolabeling of fixed samples. As a control for antibody specificity, Mad1 antibody was incubated with 15 µg of purified antigen or Bovine Serum Albumin (BSA, as control) prior to use on an immunoblot of *P. mammillata* extract.

### 2.4. Western Blot Analysis

To analyze cell cycle progression using the CDK1 substrate PP1A, protein extracts were prepared from treated embryos at 5 min intervals starting from the 2-cell stage. For each time point, we collected equal numbers of embryos (5 for *C. hemisphaerica* and approximately 100 for *P. lividus*), removed sea water, and resuspended them in Laemmli sample buffer (50 mM Tris-HCl (pH 6.8), 2% SDS, 0.1% bromophenol blue, 10% glycerol, 100 mM dithiothreitol). Protein samples were separated on 10% SDS-polyacrylamide gels and transferred to nitrocellulose membranes. After blocking in 3% BSA, to preserve phospho-antigens, membranes were incubated overnight at 4 °C with rabbit anti-phosho-PP1 antibody 52 (pospho-T320, Abcam, Cambridge, UK; 1:1000). After washing, membranes were incubated with anti-rabbit horseradish peroxidase-conjugated secondary antibody (Jackson ImmunoResearch, Ely, UK; 1:10,000) and detection was carried out with SuperSignal West Pico chemiluminescent substrate (Thermo Scientific, Waltham, MA, USA) as described by the manufacturer.

To evaluate the presence of endogenous Mad1 or Mad2 proteins, equivalent numbers of unfertilized eggs or 2-cell stage embryos of *P. mammillata* were treated with DMSO or nocodazole, lysed in Laemmli sample buffer and analyzed by Western blot. The number of eggs/embryos loaded per lane was about 100 for Mad1 and about 1000 for Mad2 which is less abundant. Membranes were incubated in 2% milk with anti-Mad1 antibody (1:3000) or anti-Mad2 antibody (1:1000), then washed and incubated with anti-mouse horseradish peroxidase-conjugated secondary antibody (Jackson ImmunoResearch, 1:10,000). The blots were subsequently reprobed with anti-tubulin DM1A (Sigma-Aldrich, 1:3000, mouse) or anti-ATP synthase NN18 [22] (Sigma-Aldrich, 1:5000, mouse) as loading controls.

### 2.5. Immunofluorescence

Eggs or embryos were fixed overnight in −20 °C 90% methanol containing 50 mM EGTA. After fixation embryos were washed 3 times in phosphate-buffered saline (PBS) containing 0.1% Triton X-100, preblocked in PBS containing 3% BSA for 1 h at room temperature, and then incubated overnight at 4 °C in PBS containing 3% BSA and the appropriate dilution of primary antibody. The mouse anti-pH3 (phospho S10, Abcam) antibody was diluted 1:1000, the rabbit anti-pH3 (phospho S10, Sigma-Aldrich) 1:2000, the mouse anti-Nup153 (Covance, Princeton, NJ, USA) 1:500, the mouse anti-tubulin DM1A (Sigma-Aldrich) 1:500, and the mouse anti-Mad1 (this study) 1:250. Following 3 washes in PBS-0.1% Triton X-100, embryos were incubated with specific fluorescently-labeled secondary antibodies at room temperature for 1–2 h. Following 2 further washes in PBS-0.1% Triton X-100, embryos were incubated for 10 min in PBS-0.1% Triton X-100 containing Hoechst (5 µg/mL), washed twice and then mounted in Citifluor AF1 (Science Services, München, Germany) for imaging and quantification. Each experiment was repeated 3–5 times and 25–200 embryos (depending on the species) were counted for each sample.

### 2.6. EdU (5-Ethynyl-2′-Deoxyuridine) Staining

5-ethynyl-2′-deoxyuridine (EdU) staining was performed using the Click-iT EdU Imaging Kit (Invitrogen), following the protocol provided by the manufacturer. Briefly, 10 µM EdU was added to MFSW once 95% of *P. lividus* embryos completed first cytokinesis (90 min post fertilization, 2-cell stage), at the same time as DMSO or drugs. Embryos were maintained in EdU for 2 generation times (50–60 min each). When control reached the 8-cell stage (around 210 min), all embryos were fixed in 3.7% paraformaldehyde/PBS for 15 min at room temperature. Following 2 washes in PBS containing 0.1% Triton X-100, embryos were permeabilized in PBS-0.5% Triton X-100 for 20 min at room temperature and washed again twice in PBS containing 3% BSA. Following a 30 min Click-iT labeling reaction, embryos were washed extensively in PBS-0.1% Triton X-100 and mounted in Citifluor AF1 for imaging. Then, 50 embryos were analyzed for each condition in 3 independent repeats.

This assay could not be carried out for *C. hemisphaerica* and *M. galloprovincialis,* as EdU incorporation was inefficient in these species.

### 2.7. Chromosome Spreads

*P. mammillata* embryos were treated with DMSO or 10 µM nocodazole for 120 min, then washed in hypotonic solution (75 mM KCl), then in 37.5 mM KCl and finally washed four times in cold methanol:acetic acid (3:1), before fixation at −20 °C overnight in methanol:acetic acid (3:1). After washing in 60% acetic acid, a few droplets of acetic acid containing the embryos were dripped onto cold methanol-washed slides from about 20 cm height, air dried, and mounted in 50% glycerol containing DAPI (4′,6-diamidine-2′-phenylindole dihydrochloride, from Merck, Darmstadt, Germany) for imaging with a Leica SP5 confocal microscope.

### 2.8. Microinjection

To observe chromatin dynamics or localization of Mad2 or Mps1 in vivo, *P. mammillata* eggs were microinjected with synthetic mRNAs encoding fluorescent proteins as previously described [19]. The H2B construct has a Venus florescent protein fused to the C-terminus of human histone 2B. The Mad2 and Mps1 constructs have a Tomato fluorescent protein fused to the C-terminus of *P. mammillata* Mad2 and Mps1, respectively. Eggs were microinjected with H2B-Venus or Mad1-Tomato or Mps1-Tomato mRNA alone, or with a mixture of H2B-Venus and either Mad1-Tomato or Mps1-Tomato, each at 4 µg/µL. After overnight incubation to allow translation, injected eggs were treated with DMSO or nocodazole, or fertilized and then treated when they reached the 2-cell stage. Eggs and embryos were imaged using either a Zeiss (Oberkochen, Germany) Axiovert 200 epifluorescence microscope or a Leica (Wetzlar, Germany) SP8 confocal microscope fitted with 40×/1.1 NA water immersion objective and 408, 488, and 552 nm lasers. Injection of mRNA encoding Venus fluorescent protein fused to either the N- or C-terminus of Mad2 gave the same results as Mad2-Tomato.

To quantify the localization of Mad2-Tomato or Mps1-Tomato proteins to mitotic chromatin in live eggs and embryos we generated Z-stack projections of the confocal planes containing DNA (generally 3 planes, 2 microns apart). Using ImageJ software (ImageJ 1.52 [23]), the red fluorescence intensity of a 7 × 7 µm square region was determined at both the location of the chromatin and a different location in the cell (three different cytoplasmic regions were averaged). The ratio of these two values (region DNA/region cytoplasm) is a measure of the enrichment of the fluorescent protein on chromosomes.

### 2.9. Time-Lapse Microscopy

Two-cell stage embryos of *P. mammillata, C. intestinalis, B. lanceolatum, P. lividus*, or *C. hemisphaerica* were placed in sea water containing appropriate drugs in glass bottom dishes (MatTek corporation, Ashland, MA, USA) or mounted between a gelatin-coated slide and coverslip using Dow Corning vacuum grease as a spacer as described elsewhere [18]. Images were acquired every 1–2 min with 20× or 40× objective lenses (depending on the size of the embryo) on a Zeiss Axiovert 200 inverted microscope equipped with Metamorph acquisition software or a Zeiss Axioimager A2 upright microscope equipped with differential interference contrast (DIC) optics and Zen acquisition software. Multiple embryos from two conditions were always filmed in parallel, acquiring a Z-stack (step size 2–3 µm) for each position.

### 2.10. CellMask Staining

For 3D reconstruction of 2-cell stage volumes following first cytokinesis, live embryos were incubated in MFSW containing 1.5 µg/mL of the plasma membrane stain CellMask Orange (Invitrogen) for 3–5 min. Embryos were then transferred to fresh MFSW in glass bottom dishes (MatTek corporation) and imaged using a Leica SP8 confocal microscope, acquiring stacks of 50–80 Z-steps (2–3 µm intervals). To measure blastomere volume the CellMask signals were manually traced and 3D rendered using Imaris software (Imaris 5.5, BitPlane, Zurich, Switzerland).

## 3. Results

### 3.1. Multispecies Survey Identifies Two Classes of Embryos with Different Mitotic Responses to Spindle Defects

**Figure 1:**
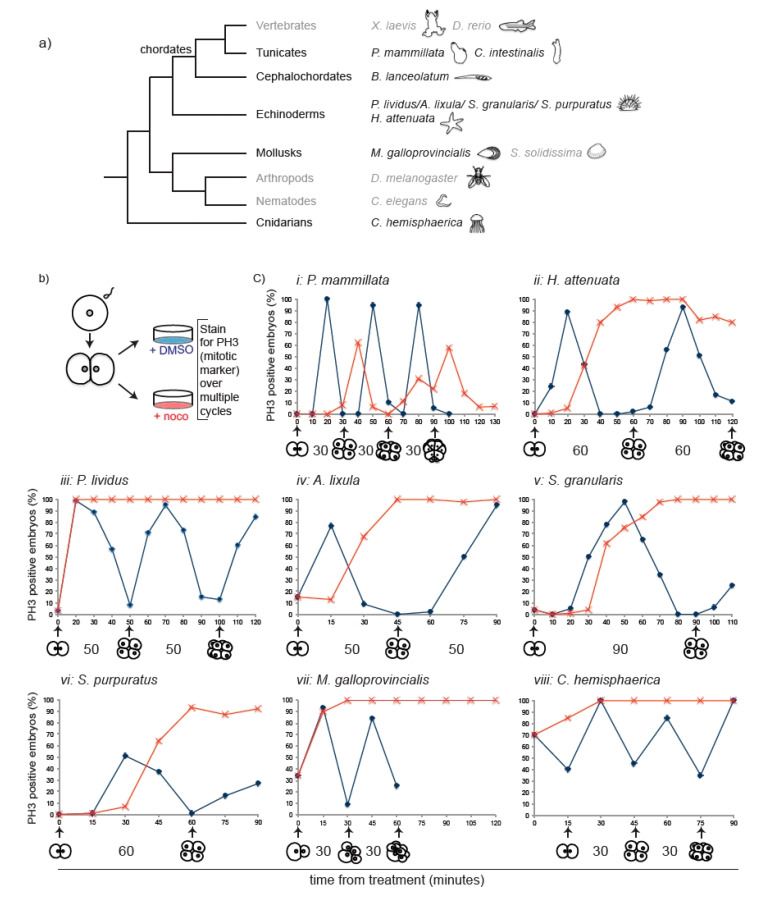
Nocodazole-induced spindle depolymerization defines two classes of embryos with qualitatively different mitotic responses. (**a**) Phylogenetic tree indicating phyla and species. Species analyzed in this study are in black, species for which information was obtained from the literature in grey. (**b**) Schematic representation of assay used to test mitotic progression. Two-cell stage embryos of selected species were treated either with DMSO or nocodazole and then fixed every 10–15 min for pH3 immunostaining. (**c**) Percentage of embryos accumulating pH3 in the presence of DMSO (blue) or nocodazole (red) over time (minutes indicated in x axis). Representative of 4 independent experiments, *n* = 50–200 embryos for each time point. Drugs were added when at least 90% of embryos were at 2-cell stage (t0). For *M. galloprovincialis*, control could not be quantified past 8 cells as divisions become asynchronous within each embryo. The duration of normal cell-cycle timing for each species is indicated under the corresponding graph.

The SAC monitors kinetochore–microtubule interactions and in somatic cells it delays mitotic progression in response to spindle defects. To assess SAC response during embryogenesis, we monitored mitotic progression in the presence of microtubule defects in 2-cell stage embryos from representative species of the main metazoan groups (Figure 1a). To complement the data already available in the literature for vertebrates (*X. laevis* and *Danio rerio*) and nematodes (*C. elegans*), we chose the tunicate *P. mammillata*, the echinoderms *Hacelia attenuata* (subphylum Asteroidea), *Paracentrotus lividus*, *Arbacia lixula*, *Sphaerechinus granularis,* and *Strongylocentrotus purpuratus* (subphylum Echinoidea), the mollusk *Mytilus galloprovincialis*, and the cnidarian *Clytia hemisphaerica*.

Different microtubule poisons can provoke variable levels of SAC activity [16]. In order to analyze SAC response under comparable conditions, we therefore performed our analysis using the microtubule depolymerizing drug nocodazole at a concentration (10 µM) that completely depolymerizes spindle microtubules (Figure S1a), generating a full complement of unattached kinetochores and maximum SAC signal. Treatment with 10 µM nocodazole after first division (2-cell stage) blocked further cytokinesis in embryos of all selected species. As histone H3 is specifically phosphorylated during mitosis [24], mitotic progression was assessed by following the phosphorylation status of histone H3 (Phospho Histone H3, pH3), as well as chromosome condensation, over the equivalent of at least one cell cycle time in both control (+ DMSO) and nocodazole-treated (+ noco) embryos (Figure 1b). We observed two qualitatively different responses to nocodazole treatment (Figure 1c). In line with previous data from frog and fish, *P. mammillata* embryos continued to cycle in the presence of nocodazole, as evidenced by pH3 oscillation (Figure 1ci) occurring concomitantly with rounds of chromosome condensation and decondensation, suggesting lack of efficient SAC activation in these embryos. However, the response to microtubule depolymerization of embryos from all other analyzed species was strikingly different. Within 10–30 min of nocodazole treatment (depending on species), embryos showed condensed chromosomes and accumulated the mitotic marker pH3, indicating mitotic commitment. Both mitotic markers were maintained for the length of time equivalent to at least one cell cycle in the presence of nocodazole (Figure 1cii–viii), and for some species, like the sea urchin *P. lividus* (Figure 1ciii), the mollusk *M. galloprovincialis* (Figure 1cvii), and the cnidarian *C. hemisphaerica* (Figure 1cviii), the mitotic arrest was extended up to 2–3 times their cell cycle duration. To exclude that the observed mitotic arrest was due to a toxic effect of the high nocodazole dose, we analyzed mitotic progression in *P. lividus* 2-cell stage embryos in the presence of different concentrations of nocodazole. A delay in mitotic progression was observed under all conditions (Figure S1b), although the length of the delay depended on the drug concentration. Thus, contrary to the generally accepted dogma that metazoan embryos lack spindle checkpoint activity, early embryos of echinoderm, mollusk, and cnidarian species significantly delay mitotic progression in the presence of spindle defects.

### 3.2. The Mitotic Delay Observed in Jellyfish, Sea Urchin, and Mussel Embryos Depends on the SAC Kinase Mps1

**Figure 2:**
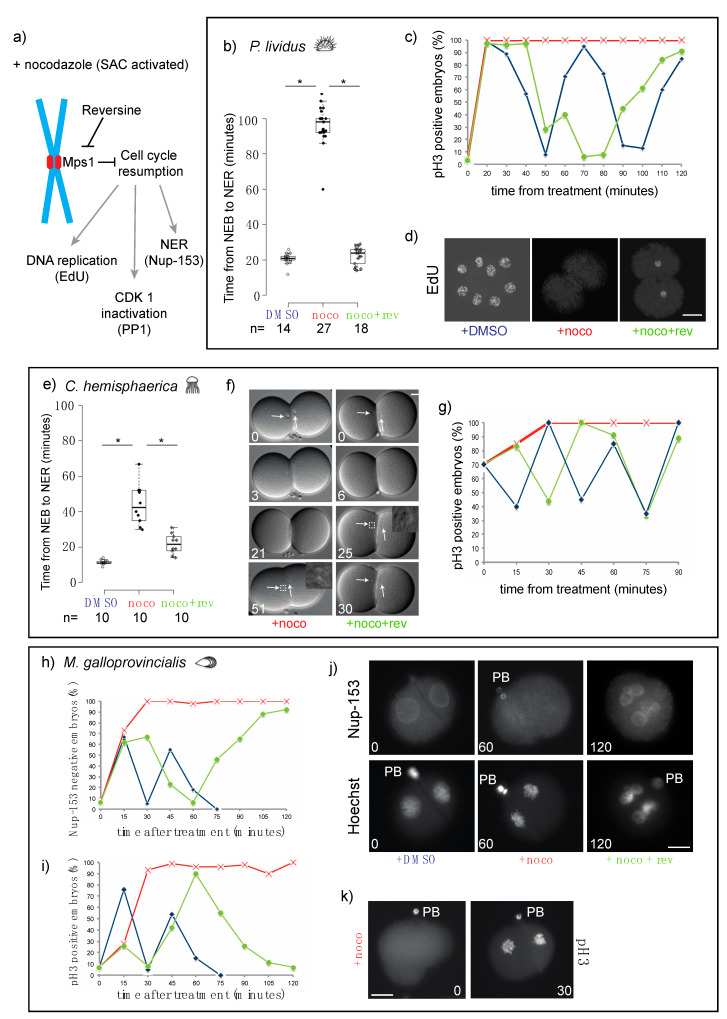
Microtubule depolymerization causes an Mps1-mediated mitotic block in cleavage stage embryos of *P. lividus*, *C. hemisphaerica*, and *M. galloprovincialis.* (**a**) Schematic representation of the effect of reversine on cell cycle progression during spindle assembly checkpoint (SAC) activation (+ nocodazole). (**b**) Quantification of duration of mitosis in *P. lividus* embryos treated with DMSO, nocodazole, or nocodazole and reversine. Mitosis was measured as time from nuclear envelope breakdown (NEB) to nuclear envelope reformation (NER). Boxes represent 25th–75th percentiles and the median is shown; whiskers mark 5th and 95th percentiles. Each dot represents one cell. Asterisks indicate statistical significance as determined by Student’s *t*-test, *p* < 0.001. (**c**) Quantification of embryos accumulating pH3 in the presence of DMSO (blue), nocodazole (red) or nocodazole/reversine (green) over time equivalent of two cell cycles. (**d**) Labeling of newly replicated DNA by 5-ethynyl-2′-deoxyuridine (EdU) incorporation in embryos treated with DMSO (left), nocodazole (middle), or nocodazole/reversine (right). (**e**) Quantification of duration of mitosis in *C. hemisphaerica* embryos treated with DMSO, nocodazole, or nocodazole/reversine. Box plot parameters as in (b). (**f**) Representative DIC images of embryos treated with nocodazole (left) or nocodazole/reversine (right). Arrows point at nuclei visible in DIC optics. White squares indicate nuclei-containing regions at time of NER, enlarged in insets. (**g**) Quantification of pH3 positive *C. hemisphaerica* embryos in the presence of DMSO (blue), nocodazole (red), or nocodazole/reversine (green). Representative of 4 independent experiments, *n* = 20–30 for each time point. (**h**) Quantification of Nup153-labeled and (**i**) pH3-labeled *M. galloprovincialis* embryos after treatment with DMSO (blue), nocodazole (red), or nocodazole/reversine (green). (**j**) Representative images of embryos stained for Nup153 (top), DNA (Hoechst, bottom), and (**k**) pH3. PB = polar body. Scale bar = 30 µm.

To confirm that the nocodazole-induced mitotic delay is due to SAC activation, we analyzed mitotic progression under conditions that compromised SAC activity. In the presence of spindle defects, the SAC kinase Mps1 binds to unattached kinetochores where it regulates the recruitment of other checkpoint components [25,26]. In somatic cells, inhibition of Mps1 activity leads to displacement of SAC components from kinetochores, checkpoint inactivation, and cell cycle resumption [25,27]. If the delay in mitosis observed in the presence of nocodazole is due to the activation of the SAC, then treatment with Mps1 inhibitors, like reversine [27], should restore mitotic timing in nocodazole-treated embryos, resulting in mitotic exit and cell cycle resumption (Figure 2a). For this analysis, we focused on a representative species from each animal group: *P. lividus* (Figure 2b–d), *C. hemisphaerica* (Figure 2e–g) and *M. galloprovincialis* (Figure 2h–k). When embryos completed first cytokinesis, we treated them with 10 µM nocodazole alone or in combination with 0.5 µM reversine and assayed mitotic progression using several markers (Figure 2a). Nocodazole alone caused an increase in mitotic index within 30 min of treatment, as evidenced by accumulation of cells with condensed chromosomes labeled with the mitotic marker pH3 (Figure 2c,g,i and Figure S2a), or of cells which lack nuclear membranes labeled with the nuclear pore component Nup-153 (Figure 2h). In nocodazole these mitotic indicators were all maintained for at least the equivalent of two cell cycle times. In all three species, reversine treatment shortened the nocodazole-induced mitotic arrest, resulting in chromosome decondensation (Figure 2j, Hoechst) and loss of chromatin associated pH3 staining (Figure 2c,g,i). In mussel embryos, mitotic exit after reversine treatment was further confirmed by nuclear envelope reformation (Figure 2h,j). In all three species, pH3-labeled chromosomes started to accumulate again at later time points, indicating that cells that exited mitosis upon Mps1 inhibition then resumed the cell cycle and entered a new mitosis. Similar results were also obtained using another Mps1 inhibitor, AZ3146 [28], further validating that the release of the mitotic arrest observed in those embryos is due to specific inactivation of Mps1 activity (Figure S2c,d).

For *C. hemisphaerica* and *P. lividus*, whose embryos are transparent, we measured the duration of mitosis in living embryos, as the time between nuclear envelope breakdown (NEB), which corresponds to prometaphase, and nuclear envelope reformation (NER), which marks the exit from mitosis, using DIC microscopy. In the presence of nocodazole, NER was significantly delayed compared to DMSO-treated embryos. In *P. lividus*, the duration of mitosis increased 5-fold, from 21 ± 3 to 98 ± 10 min, (Figure 2b), and in *C. hemisphaerica,* the interval between NEB and NER increased 3.5-fold, from 12 ± 1 to 44 ± 11 min (Figure 2e,f). Inhibition of Mps1 activity, by reversine treatment, resulted in a significant reduction of the mitotic arrest observed in nocodazole-treated embryos. Mitotic duration was shortened to 24 ± 5 min for *P. lividus* (Figure 2b) and to 22 ± 6 min for *C. hemisphaerica* (Figure 2e,f). To confirm mitotic exit in reversine-treated embryos we also evaluated the phosphorylation status of PP1, a mitotic target of CDK-cyclin B1 [29,30]. Indeed, in nocodazole PP1 phosphorylation was maintained at a constant level for at least the duration of one cell cycle (30 min *C. hemisphaerica*, 60 min in *P. lividus*), whereas SAC impairment resulted in rapid loss of PP1 phosphorylation in both *P. lividus* and *C. hemisphaerica* nocodazole-treated embryos (Figure S2b). In *P. lividus* embryos, cell cycle resumption was further confirmed by visualization of DNA replication, using EdU incorporation. DNA replication, which was undetectable in nocodazole-treated embryos over 2 cell-cycle times (120 min post treatment), resumed following reversine treatment leading to nuclear staining within 80 min (Figure 2d). Taken together these results show that in sea urchin, cnidarian, and mollusk embryos, the mitotic block caused by spindle perturbations depends on spindle checkpoint activity.

### 3.3. Chordate Embryos Do Not Arrest in Mitosis in the Presence of Spindle Perturbations

**Figure 3:**
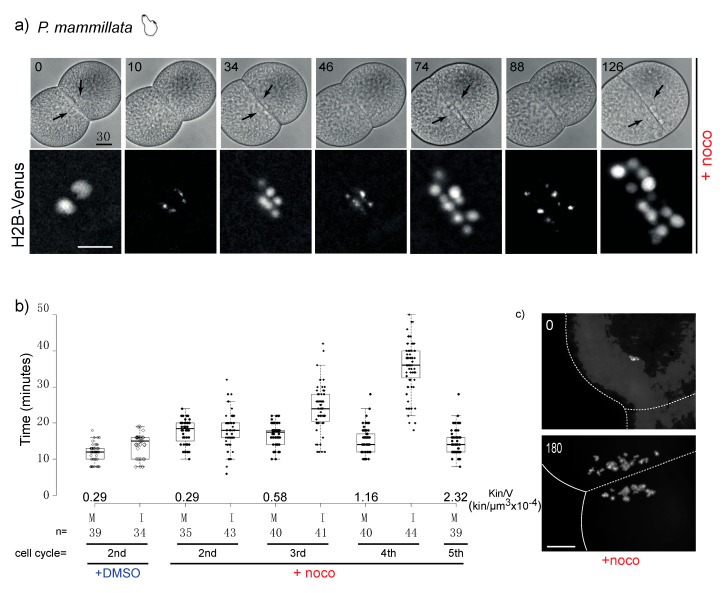
*P. mammillata* 2-cell embryos do not arrest in mitosis in the absence of spindle microtubules. (**a**) Selected frames from a time-lapse movie of a *P. mammillata* embryo expressing the DNA reporter, H2B-Venus, treated with 10 µM nocodazole after first cleavage. Numbers indicate minutes after treatment. Arrows indicate nuclei visible in bright field optics. See movie 1. (**b**) Duration of mitosis (M, NEB to NER) and interphase (I, NER to NEB) in *P. mammillata* embryos treated with DMSO or nocodazole. Kin/V indicates kinetochore to cell volume ratio at each cell cycle starting from fertilization (1st is 1-cell mitosis; 2nd is 2-cell mitosis). Box plot parameters as in Figure 2b. In nocodazole-treated embryos the amount of DNA increases due to subsequent rounds of DNA replication without intervening cytokinesis. Duration of mitosis in control DMSO-treated embryos is constant in all four analyzed cell cycles. (**c**) DAPI (4′,6-diamidine-2′-phenylindole dihydrochloride)-stained chromosome spreads from DMSO and nocodazole-treated (180 min) *P. mammillata* embryos. After 180 min nocodazole treatment, embryos have undergone 4 more cell cycles and have 4 times more DNA than at time 0. In the absence of microtubules, the nuclei which form around chromosome clusters (karyomeres) do not fuse and are dispersed over time by cytoplasmic flow (in (**a**,**c**)). Scale bar = 30 µm.

In our multispecies survey the tunicate *P. mammillata* was the only species whose embryos did not arrest in mitosis in the presence of nocodazole. We confirmed the lack of mitotic delay using live microscopy to follow nuclear behavior in DMSO- or nocodazole-treated embryos. Indeed, both control and nocodazole-treated embryos underwent multiple consecutive rounds of NEB and NER and chromosome condensation and decondensation (Figure 3a). Measurements of the duration of mitosis, as the time from NEB to NER, showed only a slight difference between DMSO and nocodazole-treated embryos (<0.5-fold). As nocodazole-treated *P. mammillata* embryos underwent subsequent cell cycles, the duration of interphase (I), measured as time from NER to NEB, increased at each cycle (Figure 3b), consistent with previous observations in *C. elegans* [15] and vertebrate tissue culture cells [31]. The duration of mitosis, however, remained unchanged, despite the increase in chromosome number and kinetochore to cell volume ratio, due to continuous cycling without intervening cytokinesis (Figure 3c). These results demonstrate that differently from echinoderm, cnidarian, nematode and mollusk early embryos, *P. mammillata* embryos do not arrest mitotic progression in the presence of spindle poisons. Thus, in *P. mammillata* embryos SAC is either silenced or very inefficient during embryonic cleavage.

Based on the analysis shown above and the available literature only the tunicate (*P. mammillata)* and vertebrate (*X. laevis* and *D. rerio*) embryos failed to trigger a mitotic delay in response to spindle defects during cleavage. Since tunicates and vertebrates, together with cephalochordates, form the chordate clade (Figure 1a), we questioned whether lack of efficient SAC activity during cleavage is a common feature of chordate embryos (excluding mammals, which have highly atypical early development featuring slow, somatic-type cell cycles). To address this question, we analyzed the mitotic response to microtubule depolymerization in *Ciona intestinalis,* another tunicate species, and in *Branchiostoma lanceolatum*, a species representative of the cephalochordate group.

**Figure 4:**
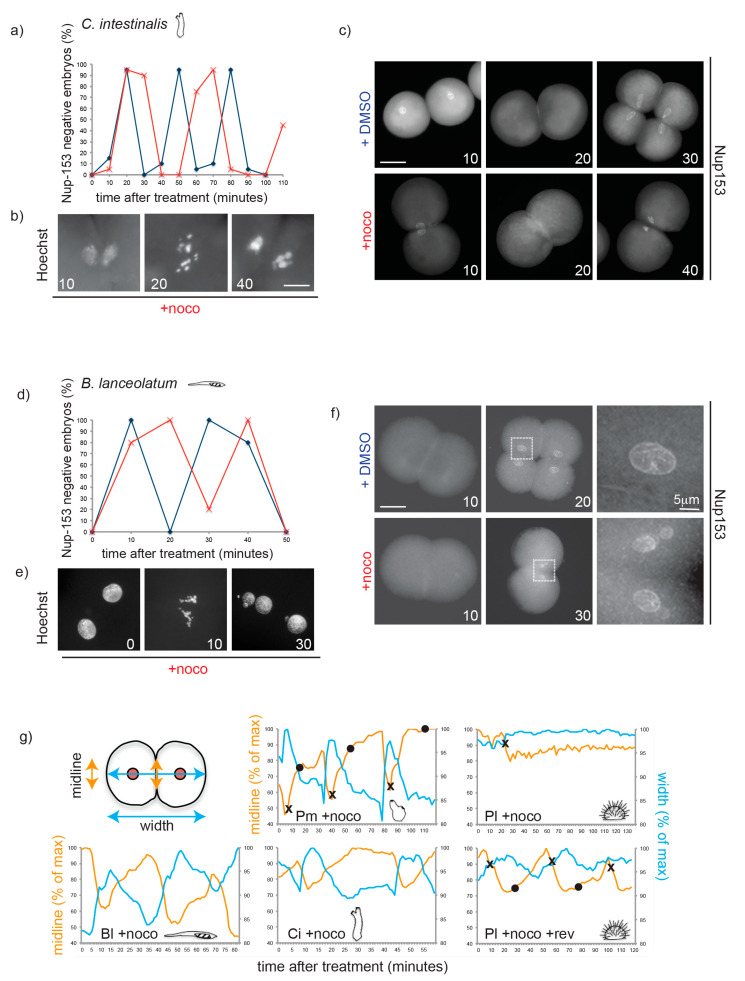
Nocodazole treatment does not delay mitotic progression during cleavage in chordate embryos. (**a**) Quantification of Nup153-negative *C. intestinalis* embryos (without nuclei = mitosis) in the presence of DMSO (blue) or nocodazole (red) over the time of 3 divisions (2–16 cells). Representative of 3 independent experiments. *n* = 20–30 embryos for each time point. (**b**) Representative *C. intestinalis* Hoechst-stained nuclei and (**c**) Nup-153-stained embryos. (**d**) Percentage of *B. lanceolatum* embryos without nuclei, as determined by Nup-153 staining, in the presence of DMSO (blue) or nocodazole (red) over the time of two divisions (2–8 cells). Representative of 3 independent experiments. *n* = 50–100 embryos per time point. (**e**) Representative *B. lanceolatum* Hoechst-stained nuclei and (**f**) Nup-153-stained embryos. Numbers indicate minutes after treatment. (**g**) Measurement of long axis of embryo (width, blue) and cell–cell contact region (midline, orange) during 2–3 cell cycles in a representative embryo of *B. lanceolatum* (Bl), *P. mammillata* (Pm, see movie 1), *C. intestinalis* (Ci), and *P. lividus* (Pl) in the presence of nocodazole (+ noco; movie 2) or for Pl nocodazole/reversine (Pl + noco + rev; movie 3). Measurements are reported as percentage of maximum length throughout the recording. Crosses (×) correspond to NEB, and circles (•) to NER. Scale bar = 30 µm, unless otherwise indicated.

As shown in Figure 4, the response of both species to nocodazole treatment was very similar to vertebrates and *P. mammillata.* Although nocodazole treatment blocked cytokinesis, nuclei of both *B. lanceolatum* and *C. intestinalis* embryos continued to cycle and underwent several subsequent mitoses, as evidenced by rounds of nuclear envelope breakdown and reformation (Figure 4a,c for *C. intestinalis* and Figure 4d,f for *B. lanceolatum*) and of chromosome condensation and decondensation (Figure 4b,e). As neither of these embryos is transparent, nuclear dynamics could not be followed in vivo. However, time-lapse microscopy revealed that these nocodazole-treated embryos underwent cyclic shape changes, whereas the SAC-arrested *P. lividus* embryo did not. As most animal cells acquire a round shape upon mitotic entry [32], we used shape change as a marker of progression through the cell cycle. Shape change was quantified on time-lapse videos by measuring two parameters (Figure 4g): contact region between the two blastomeres (midline, in orange) and total width of the embryo (long axis, in blue). In *P. mammillata, C. intestinalis*, and *B. lanceolatum* nocodazole-treated embryos, midline length and embryo width oscillated cyclically and in a reciprocal fashion. For *P. mammillata*, whose nuclei are easily visible, NEB was observed when the midline was at its shortest and the embryo width at its longest, and NER took place once cells regained full adhesion (longest midline, shortest width). In contrast for SAC proficient *P. lividus* embryos, midline length and embryo width remained essentially constant throughout the nocodazole-induced mitotic arrest (Figure 4g, Pl + noco). When SAC signaling was inhibited by reversine treatment, cyclic cell shape changes resumed with mitosis (NEB-NER) corresponding to periods of minimal blastomere contact (Figure 4g, Pl + noco + rev). Taken together these results show that embryos of *C. intestinalis* and *B. lanceolatum*, like those of *P. mammillata*, fish and frog, continue to cycle in the presence of spindle perturbations and are therefore not SAC-efficient during early embryonic development.

### 3.4. SAC Competence Does Not Correlate with Cell Size across Species

As it was previously shown that the strength of SAC response can be modulated by cell size [15], we asked whether the difference in mitotic response to spindle defects observed across species could be explained by the difference in cell size in early embryos, whose diameters range from tens of microns to millimeters depending on the animal species. We compared cell size, kinetochore number, and kinetochore to cell volume ratio at the 2-cell stage in all species used in our survey (Table 1). In this analysis, we used chromosome number as a proxy for kinetochore number.

**Figure 5:**
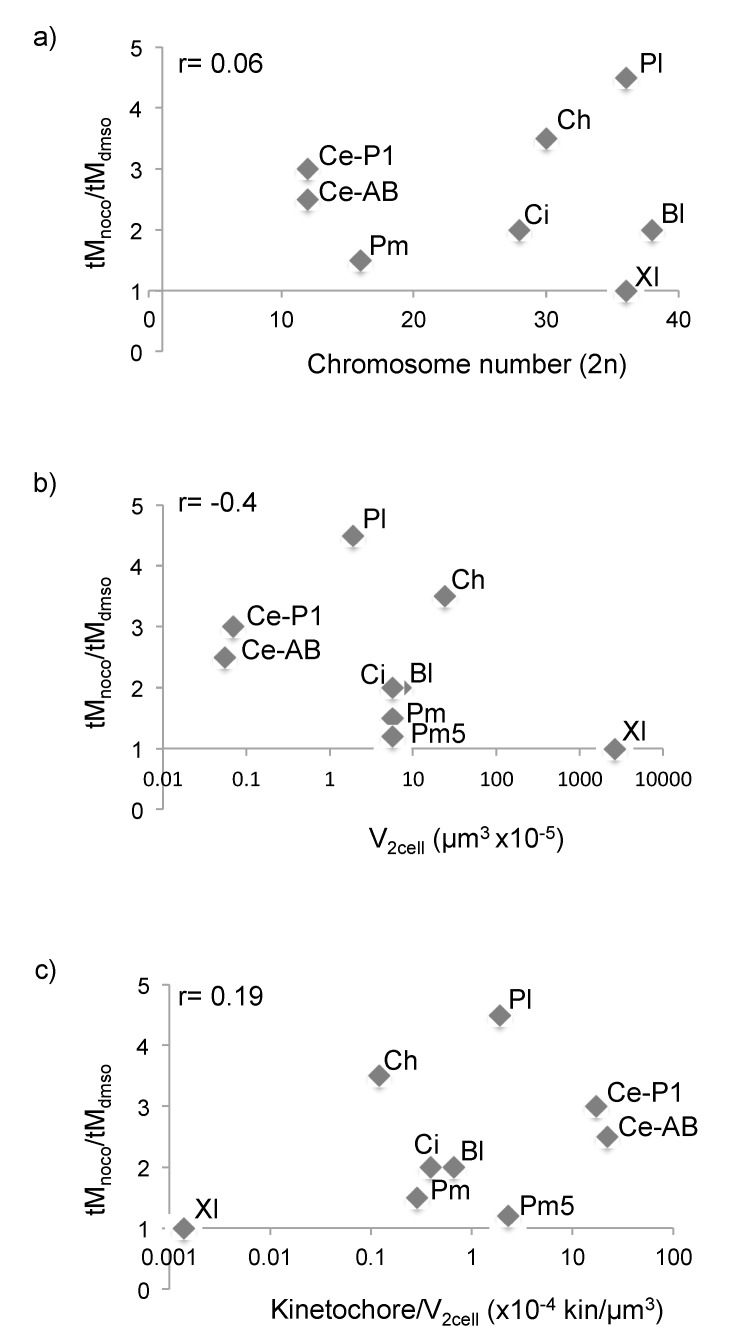
SAC response across species does not correlate with cell volume. (**a**–**c**) Ratio of average mitotic duration in nocodazole- and DMSO-treated embryos plotted against (**a**) chromosome number, (**b**) cell volume at 2-cell stage or (**c**) kinetochore/cell volume ratio at 2-cell stage. Bl = *B. lanceolatum*, *Ci* = *C. intestinalis, Ce* = *C.* *elegans*, *Pl* = *P. lividus*, *Pm* = *P.* *mammillata*, *Pm5* = ratio after 4 cycles in nocodazole from Figure 3b, *Ch* = *C. hemisphaerica* and *Xl* = *X. laevis*. For *Bl* and *Ci*, mitotic duration was estimated from the time course graphs in Figure 4a,d, as width of the curve at 50% level. *Ce* and *Xl* data were obtained from the literature. For *Ce*, as the first division is asymmetric, both cells are presented (AB and P1). r indicates the coefficient of correlation for each set of variables.

Cell volume at the 2-cell stage was calculated as half the volume of the spherical egg, except for *Mytilus galloprovincialis* whose first cleavage is unequal (Figure S3a). Cell volume was also measured empirically, by segmentation and 3D reconstruction of live 2-cell embryos stained with the membrane label CellMask Orange (Figure S3b,c), obtaining values in the same range as those calculated mathematically. Egg diameter and cell volume were both highly variable for *C. hemisphaerica*, but quite standardized for all other species (Table S1). We also included in our analysis published data for *C. elegans* [15] and *X. laevis* [5]. Comparison of the extent of nocodazole-induced mitotic delay to egg size, chromosome number, cell volume or kinetochore to cell volume ratio at the 2-cell stage (Figure 5 and Table 1) showed that the difference in SAC response across species does not correlate with any of these parameters; in fact, large cells with low kinetochore to cell volume ratio, like those of 2-cell *C. hemisphaerica* embryos, delay mitosis more efficiently than the cells of smaller embryos, like *C. elegans*, *P. mammillata*, or *B. lanceolatum*. In addition, by the fourth mitotic cycle, *P. mammillata* nocodazole-treated embryos reach the same kinetochore to cell volume ratio as SAC proficient *P. lividus* 2-cell embryos, but do not significantly delay mitotic progression (Figure 3b). Finally, the first two cells of the *Mytilus galloprovincialis* embryo have significantly different sizes but behave synchronously in our SAC response assays.

Since nuclear and spindle size also vary during development and among different cell types [45], we also checked whether SAC competence could be related to changes in either of these features. A comparison of measurements from several species showed that there is no correlation with either of these two parameters. For example, SAC-competent *P. lividus* and SAC-inefficient *P. mammillata* blastomeres have comparable sized nuclei (diameter: 14.4 ± 1 µm and 13.7 ± 2 µm, respectively) which both are smaller than the nuclei of *M. galloprovincialis,* 16.5 ± 2 µm (Table 1). Similarly, SAC-inefficient *P. mammillata* blastomeres have spindles of intermediate size between SAC-proficient *P. lividus* and *C. hemisphaerica* (Table 1). Finally, pairwise comparison of embryos with the same chromosome number (*C. intestinalis* and *M. galloprovincialis*, 28 chromosomes) showed that the variability in SAC response to spindle defects is not a straightforward consequence of the number of chromosomes or kinetochores present in a cell (Figure 5a).

Thus, our data show that cell, nuclear and spindle size, chromosome number, and kinetochore to cell volume ratio are not good predictors of SAC activity during early embryonic development.

### 3.5. Mad1, Mad2 and Mps1 Do Not Localize to Unattached Kinetochores in P. mammillata Early Embryos

**Figure 6:**
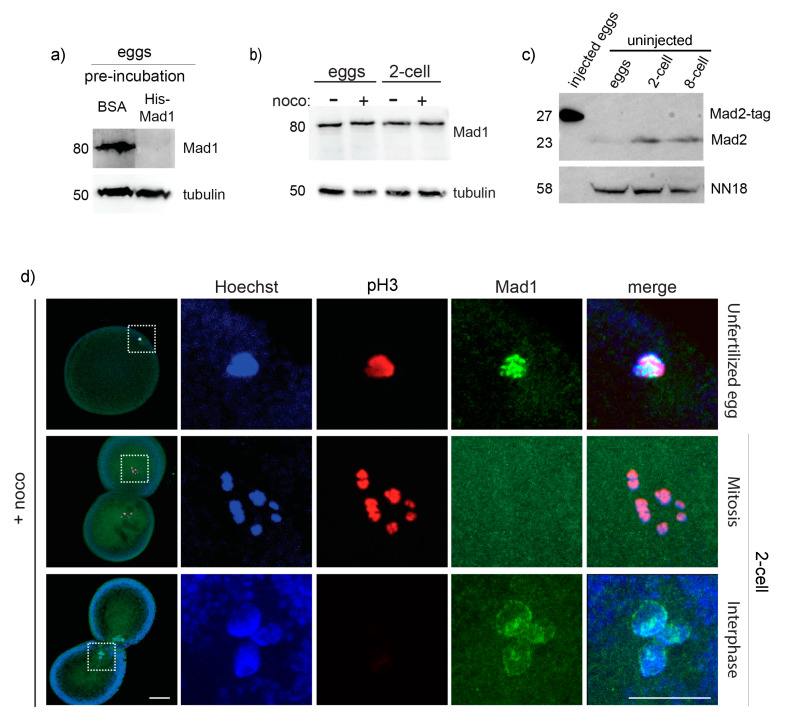
Mad1 does not localize to unattached kinetochores in 2-cell *P. mammillata* embryos. (**a**) Mad1 antibody recognizes a single 80 KDa protein in *P. mammillata* egg extract which is specifically lost (top panel) in competition assays when incubated with purified His-Mad1, but not with the equivalent amount of BSA (15 µg), whereas tubulin levels (bottom panel) are unaffected. (**b**) Mad1 level is constant in both untreated and nocodazole-treated eggs and 2-cell embryos. (**c**) Mad2 antibody recognizes a single 23 KDa protein in *P. mammillata* eggs, 2-cell and 8-cell embryos, and a larger band of the correct size (27 KDa) when tagged Mad2 protein is overproduced by microinjection (left lane: 5 eggs injected with mRNA encoding Mad2 fused to 36 additional amino acids). (**d**) Representative images of nocodazole-treated eggs (top) and 2-cell embryos (middle and bottom panels) stained for Hoechst (blue), Mad1 (green), and pH3 (red). White squares in (**d**) indicate chromatin-containing regions enlarged (5×) on the right images. Scale bar = 30 µm.

At the mechanistic level, lack of SAC activity in embryos could simply reflect absence in the egg of one or more of the basic SAC components. Although further studies are required, we do not favor this possibility since by analyzing available transcriptomic data, we determined that Mad1, Mad2, Bub1, Bub3, and Mps1 (Table S2) are expressed at the mRNA level both before and after fertilization in species that during cleavage do not delay mitosis in the presence of nocodazole, like *P. mammillata*, *C. intestinalis* (Aniseed, [46]), and *B. lanceolatum* ([47,48] and H. Escriva personal communication). Moreover, checkpoint proteins, like XMad1 and XMad2, are present in the cytoplasm of SAC-deficient *X. laevis* early embryos [49]. To test whether this is the case in ascidian embryos, we generated specific antibodies against *P. mammillata* Mad1 and Mad2. We found that the 80 KDa Mad1 protein (Figure 6a,b) and the 23 KDa Mad2 protein (Figure 6c) are present both in unfertilized eggs and in SAC-deficient 2-cell stage embryos and the level of Mad1 is not affected by nocodazole treatment (Figure 6b).

As recruitment of SAC components to unattached kinetochores is a prerequisite for SAC activation, we then tested whether Mad1 was able to accumulate at kinetochores in *P. mammillata* embryos. We observed that in unfertilized eggs, which are arrested in metaphase of meiosis 1, Mad1 localizes to discreet spots associated with the DNA upon nocodazole treatment (Figure 6d). In nocodazole-treated 2-cell stage embryos, however, Mad1 was never observed associated with chromosomes during mitosis (Figure 6d), although it does accumulate on the nuclear membrane during interphase, as previously seen in yeast and mammalian cells [50,51].

**Figure 7:**
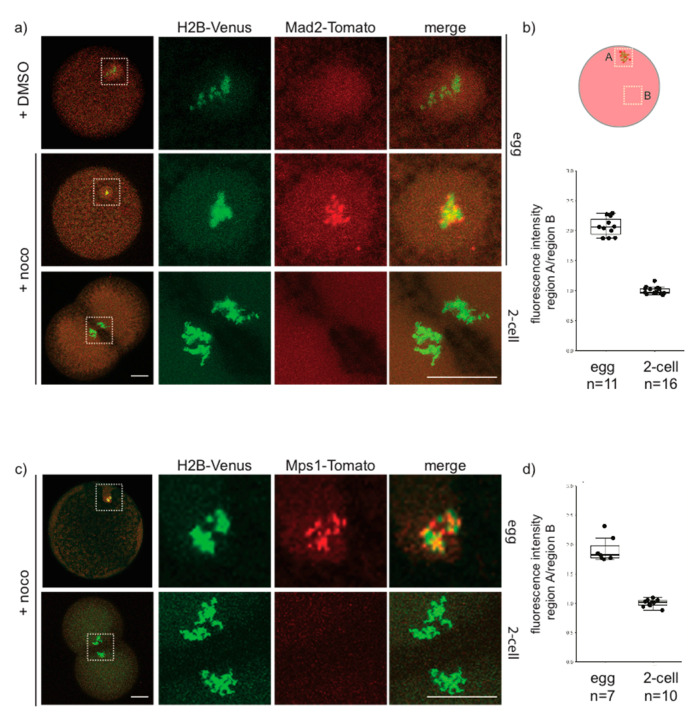
Mad2 and Mps1 are not recruited to unattached kinetochores in 2-cell *P. mammillata* embryos. (**a**) Representative images of live eggs (top, middle rows) and 2-cell embryos (bottom row) expressing histone H2B-Venus and Mad2-Tomato. (**b**) Ratio of Mad2-Tomato fluorescence intensity in chromosome region (A) and cytoplasm (B) in nocodazole-treated eggs and 2-cell embryos (**c**) Representative images of live eggs (top) and 2-cell embryos (bottom) expressing histone H2-Venus and Mps1-Tomato. (**d**) Ratio of Mps1-Tomato fluorescence intensity in chromosome region (A) and cytoplasm (B) in nocodazole-treated eggs and 2-cell embryos. White squares in (**a**,**c**) indicate chromatin-containing regions enlarged (5×) on the right images. Scale bar = 30 µm.

As Mad1 did not localize on kinetochores in early *P. mammillata* embryos, we then examined the distribution of two other SAC components: the kinase Mps1, which acts upstream of Mad1, promoting its recruitment to unattached kinetochores, and Mad2, the main MCC component. Injection of mRNA encoding fluorescently tagged Mad2 or Mps1 gave results very similar to those obtained for Mad1 antibody. As shown in Figure 7a,b, in untreated eggs Mad2-Tomato is dispersed in the cytoplasm, but it clearly localizes to discreet spots associated with the DNA (labeled with H2B-Venus) upon nocodazole treatment. However, in 2-cell stage embryos treated with nocodazole, Mad2-Tomato did not concentrate on DNA at any stage of mitosis. The same result was obtained for injection of mRNA encoding Mps1-Tomato (Figure 7c,d), which colocalized with DNA in nocodazole-treated eggs but not in 2-cell embryos. Thus, in unfertilized *P. mammillata* eggs, unattached kinetochores are able to recruit SAC components, whereas in early embryos kinetochore recognition by the SAC machinery is hindered, thus silencing SAC signaling.

## 4. Discussion

### 4.1. SAC Activity in Embryos Defines Two Classes of Animals

The spindle assembly checkpoint operates during mitosis to delay the onset of anaphase under conditions that could otherwise compromise accurate chromosome segregation [52] and is thus important for cell and organismal viability. Despite this essential function, it has long been thought that the SAC is inefficient in early development of animal embryos with large eggs, as they undergo fast cycles. Here, we have undertaken a rigorous survey of the SAC response to spindle defects in embryos of diverse animal species, combining both new experimental data and previous findings from the literature. Since different microtubule poisons can provoke variable levels of SAC activity [16], we included in our analysis only studies performed using the microtubule depolymerizing drug nocodazole at a concentration that completely depolymerizes spindle microtubules, therefore generating a full complement of unattached kinetochores and maximum SAC signal. Our analysis shows that in the presence of unattached kinetochores, mitotic progression is unperturbed in fish, frog, amphioxus, and ascidian embryos, whereas sea urchin, mussel, jellyfish, nematode and insect embryos delay mitotic exit. We conclude that there is no inherent incompatibility between the fast division typical of cleavage stage embryonic development and spindle checkpoint activation.

### 4.2. SAC Activity in Relation to Kinetochore and Cytoplasm Content

Variations in the duration of mitotic delay induced by SAC activation have been reported previously in several cellular contexts and were partially attributed to differences in cell size and kinetochore to cell volume ratio [15,17,53]. However, our analysis shows that SAC competence during embryo cleavage cycles does not correlate with reduced cell size across different species, with large jellyfish (diameter 210 µm) and starfish (240 µm) embryos mounting a prolonged block from first division and the smaller ascidian (130–140 µm) and amphioxus (130 µm) embryos not delaying mitosis but rather continuing to cycle in the absence of microtubules (Table 1). Likewise, pairwise comparisons also suggest that chromosome number (*P. lividus* and *X. laevis:* 36 chromosomes; *M. galloprovincialis* and *C. intestinalis:* 28 chromosomes) and kinetochore to cell volume ratio (*P. lividus* and *B. lanceolatum*) are not strong indicators of SAC competence at the egg-to-embryo transition across metazoans (Table 1). Consistent with this conclusion, it was previously reported that in *D. melanogaster*, whose eggs are 500 µm long and have only four chromosome pairs, treatment of stage 3–6 syncytial embryos with colchicine arrests the nuclear cycle at a prometaphase-like stage, suggesting that the SAC is active from early cleavage stage in these large insect cells [41,54]. However, in early *Drosophila* embryos cyclin B degradation and CDK inactivation occur only locally [55] in the area of the spindle rather than at the level of the whole embryo. This observation raises the possibility that the SAC may be regulated locally in the vicinity of the chromosomes. Indeed, previous work carried out in PtK_1_ cells containing two separate spindles showed that once all kinetochores are attached to spindle microtubules within one spindle, anaphase will start irrespective of the presence of unattached kinetochores on the second spindle, suggesting that the SAC signal is not diffusible [56]. An alternative possibility is that the viscosity of the cytoplasm is discontinues in the cell, interfering with long-range diffusion of the SAC signal away from the spindle region. In both hypotheses, if SAC action is limited to the spindle region then the strength of the SAC response may be a function of the volume of a subcellular region local to the spindle area, rather than total cell volume. Spindle size itself, defined as pole to pole distance in metaphase, however, was shown to scale linearly with cell size across embryos of many different species [45] and we confirmed this trend for our species. Thus for 2-cell embryos, difference in spindle size is unlikely to explain the difference in SAC activity observed across same-sized embryos. Similarly, we show that SAC strength does not correlate with changes in nuclear volume. We can conclude that there is no straightforward link between any of the cellular parameters that we analyzed, which include kinetochore number, spindle length, volume of cytoplasm or nucleus, and the categorization of embryos into SAC-proficient and SAC-deficient.

Recent work carried out in *C. elegans* embryos showed that SAC strength is influenced by cell fate [57]. This finding suggests that the variability in SAC activity may instead be related to differences in developmental strategies and to the establishment of cell lineages during cleavage. Based on our current knowledge of cell fate specification in different species, however, this does not appear to be the case, as SAC competent species include animals with clear segregation of developmental potential at the 2-cell stage, such as *C. elegans* and *M. galloprovincialis*, as well as animals whose first two blastomeres are identical with respect to cell fate, such as *P. lividus* and *C. hemisphaerica*. We therefore favor the hypothesis that SAC is silenced during early development in some metazoan embryos, in a manner independent of specific cellular attributes like cell size, kinetochore number, and cell fate, whereas those factors probably do influence SAC strength once the SAC becomes active in a given species.

### 4.3. SAC Deficient Embryos as an Evolutionary Novelty in the Chordate Lineage

While we could not uncover any physical explanation for variation in SAC efficiency, it was immediately apparent when looking at their phylogenetic grouping that all species with SAC-deficient embryos are chordates, whereas species in all non-chordate clades possess SAC-competent embryos. Thus, based on our analysis we can propose that SAC proficiency in early development is an ancestral feature of metazoans and that loss of the SAC in cleaving embryos may be associated with the emergence of the chordate lineage. Sampling a wider number of species and metazoan groups under these same experimental conditions will be required to test this hypothesis further. Some supporting examples of non-chordate species being SAC positive can already be inferred from the literature, although the use of different drugs and assays to assess mitotic progression complicates comparisons. As already mentioned, colchicine treatment blocks cyclin B degradation in clam embryos during first mitosis [13], and addition of nocodazole blocks nuclear division in embryos of another mollusk, the gastropod *Ilyanassa obsoleta* [58]. Similarly, treatment with colchicine delays nuclear division at least for the length of one cell cycle in embryos of the fruit fly *D. melanogaster* [41] and in binucleated embryos of the gall midges *Wachtliella periscariae* [59] and *Heteropeza pygmaea* [60].

Given our current understanding of SAC function in maintaining ploidy, it is hard to understand what selective advantage could be associated with loss of SAC signaling in chordate embryos. At this point we can only speculate that SAC silencing is a by-product of some other change in reproductive regulation or oogenesis that could impact the levels of mitotic molecular regulators or the availability of kinetochores. Based on our work we favor this second hypothesis. Unlike SAC competent embryos, like those of *C. elegans* whose unattached kinetochores can recruit SAC components [15,61], Mad1, Mad2 and Mps1 do not accumulate on unattached kinetochores in SAC-deficient *P. mammillata* early embryos.

Thus, we suggest that in SAC-deficient embryos a modification of kinetochores or of the checkpoint machinery has occurred, which interferes with the recognition of unattached kinetochores by the SAC components and the generation of the inhibitory signal. As Mps1 is one of the most upstream components of the SAC signaling cascade and one of the first SAC proteins to be recruited to unattached kinetochores, and its kinase activity is required for the recruitment of both Mad1 and Mad2, it is likely that regulation of Mps1 localization is critical for the change in SAC activity observed in chordate embryos. However, Mps1 kinetochore localization appears to be very complex and its control remains still poorly understood. It is therefore difficult to predict the mechanism that underlies the different pattern of Mps1 localization in chordate eggs and embryos. Mps1 localization at kinetochores was shown to occur through direct binding with the kinetochore components Hec1/Ndc80 and Nuf2 [62]. As both these proteins are also required for kinetochore binding to spindle microtubules it is unlikely that the switch in Mps1 recruitment is modulated by changes in their abundance, but more likely by posttranslational modifications that interfere with their interaction. Interestingly, it was previously shown that MAPK (mitogen-activated protein kinase)-dependent phosphorylation of Mps1 is essential for its kinetochore localization and for checkpoint function in *Xenopus* egg extracts [62]. MAPK, which is active in unfertilized eggs, is inactivated following fertilization to allow cell cycle resumption [63], concomitantly with loss of Mps1 localization in *P. mammillata* embryos. Sequence analysis, however, shows that S281, one of the MAPK phosphorylation sites in Mps1 [64], is conserved in all species irrespective of their SAC response, whereas S844 [65] is missing both in SAC-efficient *P. lividus* embryos and SAC-inefficient *P. mammillata* embryos. Further analyses will be required to understand the underlying molecular mechanism controlling spindle checkpoint silencing during early development in SAC-deficient embryos and the possible links between these changes in mitotic control and evolutionary transitions.

## Figures and Tables

**Table 1:** Morphometric data for embryos of all analyzed species.

	Species	Oocyte Diameter (µm)	Chromosome Number (2n)	Kin/V_2cell_ × 10^−4^ (kin/µm^3^)	V_nucleus_ (µm^3^)	Spindle Length (µm)	SAC Competence Reference
Chordates	*Xenopus laevis*	1000	36	0.0014	3053 [33]	53.5 [34]	− [5]
	*Danio rerio*	800	50	0.0037			− [6]
	*Ciona intestinalis*	140	28	0.39			− this study
	*Phallusia mammillata*	130	16	0.29	1345	20–30	− this study
	*Branchiostoma lanceolatum*	130	38 [35]	0.66			− this study
Echinoderms	*Hacelia attenuata*	155	44 [36]	0.45			+ this study
	*Paracentrotus lividus*	90	36 [37]	1.9	1562	18 [38]	+ this study
	*Arbacia lixula*	80	44 [36]	3.3			+ this study
	*Strongylocentrotus purpuratus*	80	42 [39]	3.1			+ this study
	*Sphaerechinus granularis*	110	42 [36]	1.2			+ this study
Mollusks	*Mytilus galloprovincialis*	65	28 [40]	Small = 6.6 Large = 2.5	2393		+ this study
	*Spisula solidissima*	60	28	6.7			+ [11]
Arthropods	*Drosophila melanogaster*	500	8	0.018			+ [41]
Nematodes	*Caenorhabditis elegans*	50	12	AB = 22 [15] P1 = 17 [15]	523 [42]	13 [43]	+ [44]
Cnidarians	*Clytia hemisphaerica*	210	30	0.12		30–35	+ this study

## Supplementary Materials

The following are available online at https://www.mdpi.com/2073-4409/9/5/1087/s1, Figure S1: Effect of nocodazole on spindle organization and mitotic progression, Figure S2: Mps1 inhibition releases the nocodazole-induced mitotic block observed in 2-cell embryos of *P. lividus* and *C. hemisphaerica*, Figure S3: Measurement of volume in 2-cell stage embryos, Table S1: Values associated with Table 1 and Figure 5, Table S2: Sequences of analyzed SAC components, Table S3: Values associated with Figure 2, Table S4: Values associated with Figure 3, Video S1: *P. mammillata* 2-cell embryos expressing H3B-Venus, in the presence of 10 µM nocodazole, Video S2: *P. lividus* 2-cell embryos in the presence of 10 µM nocodazole, Video S3: *P. lividus* 2-cell embryos in the presence of 10 µM nocodazole and 0.5 µM reversine.
