# Supplementary material for: The Spindle Assembly Checkpoint Functions during Early Development in Non-Chordate Embryos
Source: Cells. 2020 Apr 28;9(5):1087. doi: 10.3390/cells9051087 (PMC7290841; doi:10.3390/cells9051087)
Supplement: Supplementary file 1 [file cells-09-01087-s001.zip › ChenevertSupplementary/Chenevert_FigureS1.pdf]

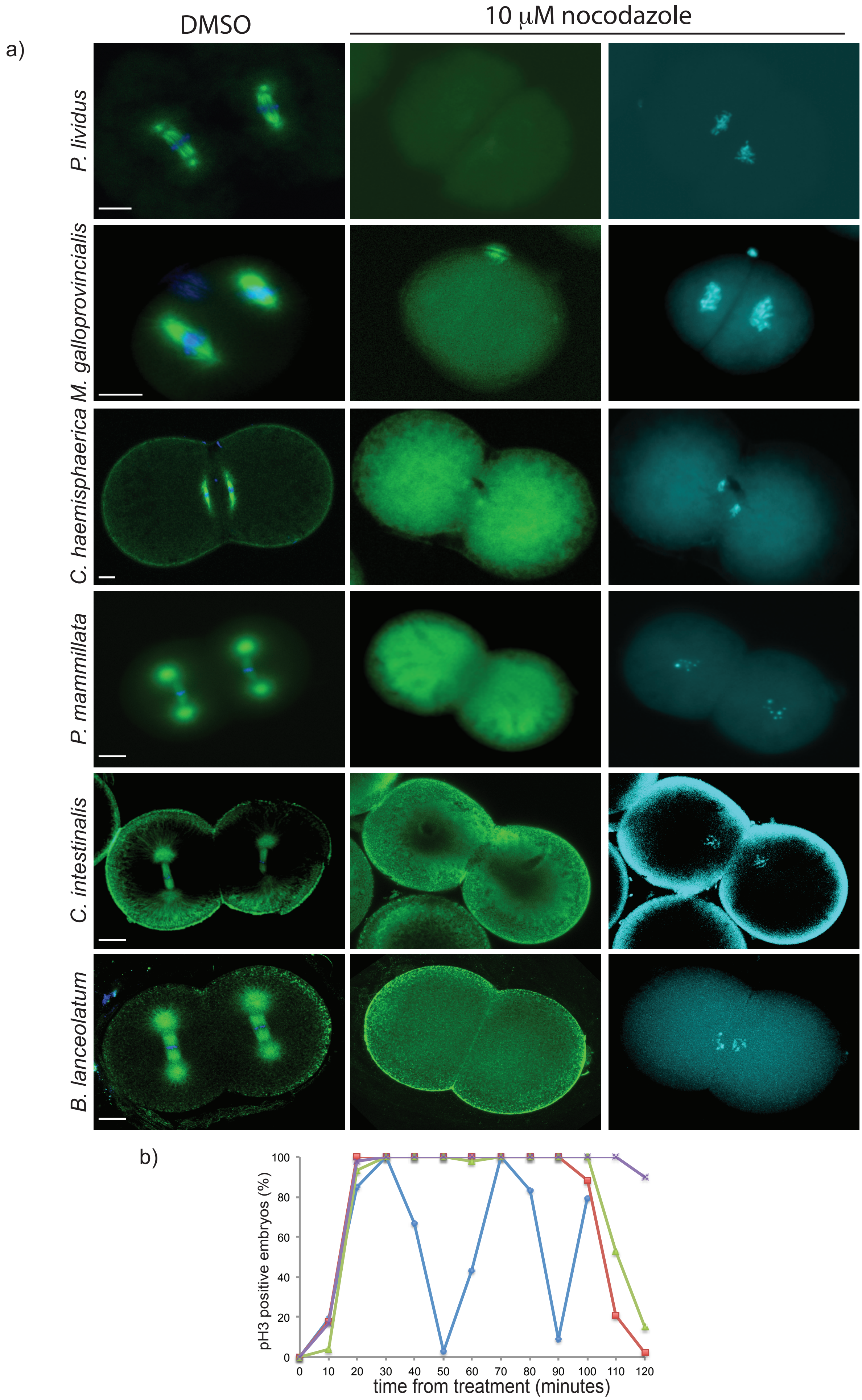

Figure S1: Effect of nocodazole on spindle organization and mitotic progression.
a) 2-cell stage embryos fixed and stained for microtubules (anti-tubulin, green) and for DNA (hoechst, blue), after 15 minutes incubation in DMSO, or in 10 μM nocodazole. Representative embryos in metaphase stage are shown. b) Quantification of pH3 positive 2-cell *P. lividus* embryos treated with DMSO (blue), 10nM (red),100nM (green) and 1μM (magenta) nocodazole. Scale bars are 30μm.
