# Supplementary material for: The Spindle Assembly Checkpoint Functions during Early Development in Non-Chordate Embryos
Source: Cells. 2020 Apr 28;9(5):1087. doi: 10.3390/cells9051087 (PMC7290841; doi:10.3390/cells9051087)
Supplement: Supplementary file 1 [file cells-09-01087-s001.zip › ChenevertSupplementary/Chenevert_FigureS2.pdf]

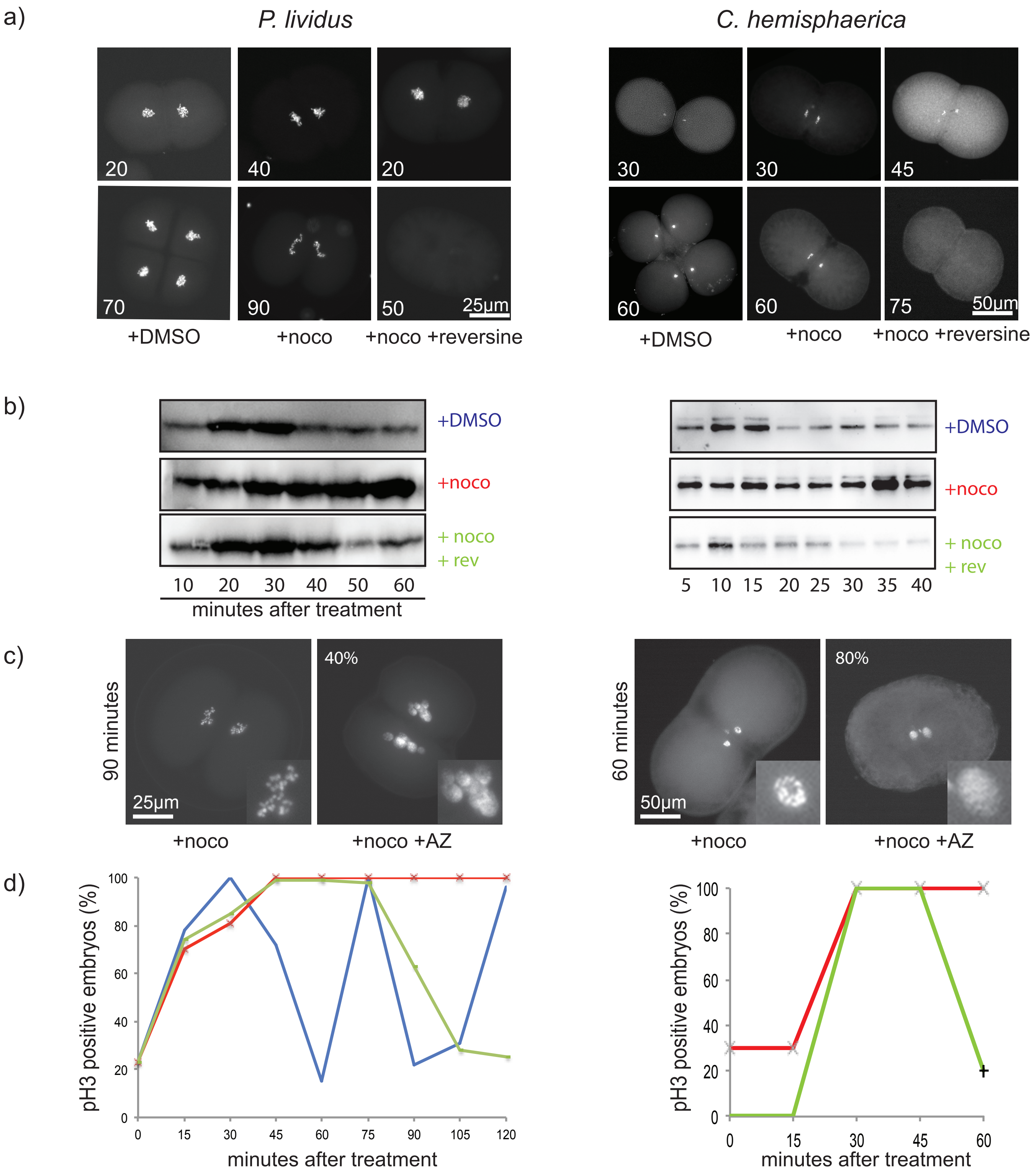

Figure S2: Mps1 inhibition releases the nocodazole-induced mitotic block observed in 2-cell embryos of *P. lividus* (left) and *C. hemisphaerica* (right).
a) Representative images of pH3 stained embryos treated with DMSO, nocodazole or nocodazole+reversine at different time points (indicated in minutes). b) Western blot analysis of phospho-PP1 in embryos treated with DMSO (top), nocodazole (middle) or nocodazole+reversine (bottom). c) Representative images of nocodazole and nocodazole + AZ3146 treated embryos stained for DNA (Hoechst). At the time point shown, 100% of nocodazole treated embryos have condensed chromosomes, whereas decondensed chromosomes accumulate upon AZ3146 treatment. d) Percentage of embryos accumulating pH3 in the presence of DMSO (blue), nocodazole (red) or nocodazole + AZ3146 (green). Representative of 3 independent experiments.
