# Supplementary material for: The Spindle Assembly Checkpoint Functions during Early Development in Non-Chordate Embryos
Source: Cells. 2020 Apr 28;9(5):1087. doi: 10.3390/cells9051087 (PMC7290841; doi:10.3390/cells9051087)
Supplement: Supplementary file 1 [file cells-09-01087-s001.zip › ChenevertSupplementary/Chenevert_FigureS3.pdf]

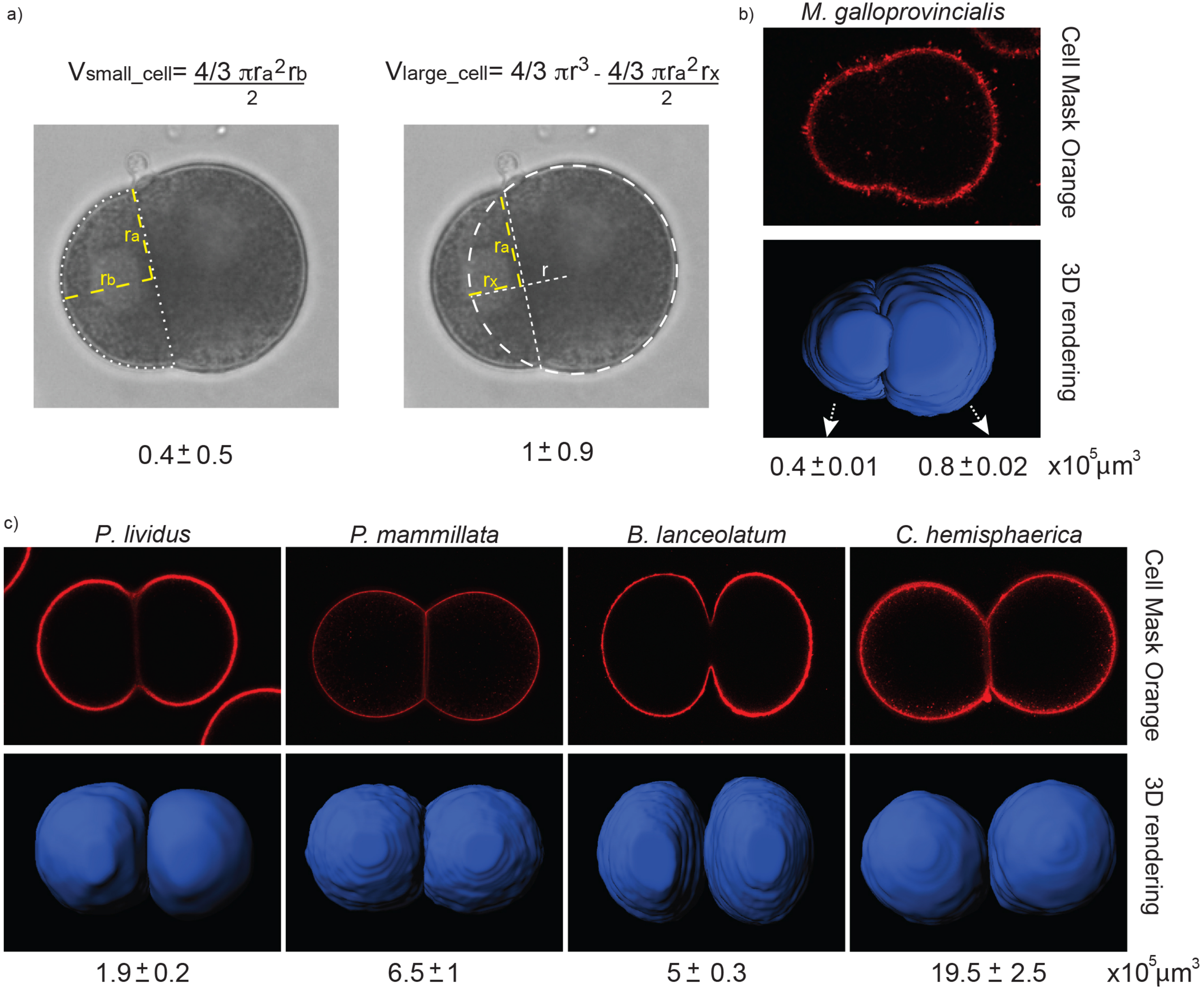

Figure S3: Measurement of volume in 2-cell stage embryos.
a) Mathematical calculation of cell volume in *M. galloprovincialis*. Average calculated volume is reported below each panel. b-c) Cell Mask Orange staining and 3D rendering of live 2-cell stage embryos of indicated species. Volume of 3D reconstruction is reported under each panel.
