# Supplementary material for: The Spindle Assembly Checkpoint Functions during Early Development in Non-Chordate Embryos
Source: Cells. 2020 Apr 28;9(5):1087. doi: 10.3390/cells9051087 (PMC7290841; doi:10.3390/cells9051087)
Supplement: Supplementary file 1 [file cells-09-01087-s001.zip › ChenevertSupplementary/Chenevert_TableS2.pdf]

S2 Table: Sequences of analyzed SAC components

Phallusia mammillata:

Mad1:
phmamm.CG.MTP2014.S241.g05985
MKVSVDNTDLSNTFFFMFLVLACGFEHDVNSRQLRNTNLSGTSNTIKENTSKASDVMMRV
KMKQIEAAHASSERAHVKANIELESKFEQLTAQNKVLKDKADSLQGKVNSLTTKLLDMQD
EVKQMRKDKEAEISKWENSYLHLETLKQEADSRLSEEMMMVTSQNQQLSEHNLMLQSQNN
ILNLKCEEHYTHMDQYKRSLEASQTVLKDYQETKSKLARSEQQVNRLKQELATLQDTKTL
AKTLKEEMNRLRHIEVEFKKIHEENYLLRQNNDNFALLQEKLHSVESKLARAELQCANVP
SLEVENNMLTQRIEQLQNMSVTKNEHSSLQIELSSLSQKLSTAQEENLNLKAELSTLRNH
NIELNSSIKAIDQKLTEKSEICASLNAQLLRLRKRAGLLAKERDSIREILQSYDAELTMT
SHTTQLNKRIENEAASNKRLYHRIEELEDENKKLAEEAMKNRLGIKTLESQKELKETPDS
PMVSQTMNEENSVLREKIQKYEAERPTLIEKIEQLEAWIEQGKIKGDYNPENTKVIHFAM
NPADLAHQKSKQDVANLKEECIRLRQKLREAADGHEVSVAEVESLKLAQEEASRAELRNQ
RLKEVFTKKIQEFRQACYSLTGYRIDTLNDSQFRLVSIKAAETELCTDERERRNEVIGDG
ILNYSDGIGEFALASPKQYSNVFKCYFSESFQSTNPGIAASSKGCKF

Mad2:
phmamm.CG.MTP2014.S64.g02288
MAAAKQLNKITLKGSAAIVSEFFFYGINNILYQRGVYPPEMFKQEKKYGLTILVSTDETL
LTYLNDNVLPQLQEWLEKGAVKRLVVVIRESETNETLERWQFEIQCESDKSENGKPATKS
KETINSEIRAVIRQVTATVTFLPLLECPCSFDLLFYTDKNLEVSEKWEDSSPCLIPNSEE
VKLRSFSTSVHKVDGAVSYKFSM

Bub1:
phmamm.CG.MTP2014.S849.g12351
MPLTNTGASVLTDSVYGGDNEVSEINGMFQMPLTNTGASVLTDSVYGGDNEVSMMPLAEP
VDKPAFEIFCDDETDEQQMPLKFNIFKDDDKSEDSENIPTPGYVSPPKRHNLSGILQPAV
GFELADPDEDSQEEEDDNIVGVEPLLTVEPAPTFLDDVTIAAGQNLPGETNLPLNQLQDI
PCTSSNNLTNSYQANRSAMSTTNKFDDSIQINFGNRNKISAESTTYWQGSAIHDVTKQDV
IEKTVNPPQEVTSEANCTEIGNQQQGVKKLSPILEASYEYEKSVQSRSRLTSSETDGQAS
VAITTTGKSFIHPPTINRKPLGELSAITYDPMKTSTAPTMYDMEPEFSSFMPHQGISKLQ
LHNDNDETTTSYSPMCLPVLNNTSIHPIELNPDAIMEDELCDQMSTSLQIAPPPDHSVFD
VTTFLSETNNLTVSGRLTIVEDPWDEELLNRLLPPQFAKNLHVIHGKTPVARKGSTLNLG
DTTYHLADMLGRGGFAKVYKATVEGKSNTIAAVKIQSPPHIWESYIVSEAKRRCNSDSFR
SSLLQIHATSVFPEASFIVSEFLSGGTLLEFVRDCAVHSRVIDDIDITFKVMKIVNSLHE
AGIIHGDIKPDNFMVVSERDPCKGRLGPLAPVLKLIDFGRAIDMKAFPAGTAFKKNCGTS
GFVCSQMMDKLPWNYHIFKLDSFQQTDFHGLAGTIHVVLYNCYMTIIKQNSGEWSITKSF
PRGNRHLWSSFFKSLLNVSTPKDELPFVDSPLPNLLQLFGEAAT

Bub3:
phmamm.CG.MTP2014.S36.g01423
MSNEFKLNECPKDGISSVKFSPSTAQFLLASSWDVSVRLYDITENTCRFRYDHKAPVLDC
CFSDSVHAWSGALDGSLLMYDFNMGRESLAGMHNAAIRCVEYCSETNVIATGGWDETVKL
WDPRNKSSIGSYSQPGKVYTMSVCGHRLIVGTSGKSVVVWDLRNMGYVEQRRESSLKYQT
RCIRSFPNKQGFVLSSIEGRVAVEYLDPSAEEQKKKYAFKCHRIKEDGIERIFSVHTIAF
HNRYNTFATGGADGFVNMWDGFNKKRLCQFHRFPAPVSSVAFSDDGSVLAVAASPLYSSD
LEPNRDVEDAIFIRHVTDAETKPK

Mps1:

phmamm.CG.MTP2014.S420.g08523
MFKENECVAESDKENTNTLNVTGKVTSNLKEVGDDPEKWNQILQDCLKLDNTLHKKRMLK
QFFLQAKKNIDPAVHRRSETYARILAQEALFISLSSRQDAGVAFRFATDTCRPVPFIHVA
FAQYEVDSGRLEKAKKILELGKMVLPDSKEISDAIKRLKSNETNLGLKNDPMFNSSVLRD
NTNSMSTPKVAKYDFGDSIMKIFPTSKHSLTDTCIKSPIRPAHIKRELEALDFPESPRSS
VTKPSFFGITTPSPPRLSFSVTKSLANRHALQTKQEHLTTSVQKPMMTSKTNGQRSGPVN
KPRRIPCALQPENDQKHKSEYPMVFPLASEVGATPTDNRIEKVFPTQPVSQVSASPTRPT
FPPPIRQPQPNLAAHAIKTDEIASWMNNVQVLRIHHKSYIVLKMIGEGGSSKVFEVFDVA
AKQIKAVKHVSLKNCDAAVKKGFLDEVKFLEQLRNNPNIVHLYTYELTGDDLYLVMECGS
TDLSKSLKRNNGRLEPYEVWYFWKKMLAALNTVHQHGIIHLDLKPANFLIVKGTLKLIDF
GIANSIQSDVTSVFKDTMVGTLNYMAPEAIVDMSSGGDGNDLKFKISPRADVWSLGCILY
LMMYGRTPFQHINHQIRKLSAITNPNTRIEFPQYGDKRLVQIVQSCLMRDAKRRPTVEQL
IKHS

Ciona intestinalis:

Mad1: XP_002126990.1 (Gene model: KH2012:KH.C9.233)
MEQRFALGMTPDNTKVMRIMGEFHDFMSAAKENSMQQSSHISAIPFSLPSDDDHKQSTALQV
SNVLSKVKLKQLESNHSSSEKLLIQRTVNLESKIKELEMEKQILHERCSKYQVQVTDLTQQS
IDLSDKVKDLEQTKHEEILKWEESAMEIQRLKQQNEQKFAMQTQMLNERINDLMSQKLTMEK
EEQLFEINQAELCSQVEQYKSQAQAAYQLQAKYDEKTNQVLQAEHTIHELKMRLENVQEAEN
MAHILKQDMNRLRQLEIDVSHLSRENSNLKLNQENCALLKEQLIAANTKLQRLEEKCNEIPK
IVAENEALKEKLNKTQNATTANVDDNVSLLQNHSSFNAKLEKEIETLKEQLATSKSRSLEDR
KNAAEYEIKFTEQTEAISSLKAQLIRLKKRASLFAYERDSIRSLLQTYDAELTMTSHTTQLN
KRLDNMTSVNKKLHDRIVELELESQRHVEDTLRHKLQVKQMQLGGSLSSGQKQEEMSISSVL
EEKSNEVLALKEKITSFETERTNLMEKIANLEAWIEQRNLNGDYNPDKTKVLHFTMNPADLA
HQQSKRDITELKEQNAKLQLKLRQLEEGHEVSMSEIEFSKEAKTKLNAAELKNQRLKEVFSK
KIQEFRQVCYSLMGFQVVCSSDGKFKLLSMYADSETDCLEFEVKSSGEIELLETEYTKTLTD
LISLHLHHQNSIPMFLSALTVNLFGQQTMMAD

Mad2: XP_002131760.1 (Gene model: KH2012:KH.C14.279)
MASNKQLNKITLKGSAAIVSEFFFYGINNILYQRGVYPPEMFKQEKKYGLTILTTTDPNLLT
YLNENVLPQLTEWIEQGVVKRLVVVIRECETNETLERWQFEIECQSDGKENSNPKSKDISVI
NSEIRAVIRQITATVTFLPLLEVPCSFDLLFYTNHDLVAPEHWEDSSACLINNSEVVKLRSF
STSVHKVDGAVSYKFSM

Bub1: XP_026692557.1 (Gene model: KH2012:KH.C11.241)
MSNEITWEVCKENVQPLRHGRNVSYLNASLQTSDEISHSLMKQKKMLEEEILTDGNLHDPID
PWDRYFKWSQQHFPEGKEDLKNFLQKYIVKFQNSDRYRNDPRYVNAWLTMSQIHDDAPTTFA
YMKSKSIGINCASFYIMWAEELEKSGNIKKAHSIYELGEENDAEPTELLSKMRNAFQLRAAR
SISTKLNENEDDKNKSELDSRRQRQALGSLDGRGKHKVLGTTRIGNTTAGVVRSQPRTSFKE
NRSSTKFKIFSEDENNEQHCVGNFASMPNNQINSKENTTAPSVWKGAEVQLNRNKTTTAISS
SNKPFTICQDVDVPSQEQATPLASRKLSKSVEVILTERKWKKHEESDFHRAIREQHGDADHN
VVRMYPVEKVYSAVGEFQPEEILAACWLKKQREEEEQKRLQRQLEEQRKQIVESERREIEAQ
RAYDDKVNQLKHREQQLRNLLQLFKDKEMQIEDKVEMVKEEQEMTIALHKVCSQLQHMQQKN
NIVEVATSSQAIQGGKSLLEDEVMECKPSSPTVCTKEAMGEIFGMFQKPLNTDVNVTKHEPS
MMQQSQFSIYCDAEMKDKTPVKFDIYEDASDNSENIPTPEYKQAPKREGLSGILQPAVGFKL
EEDDFDDDGKDEDERLFDDVYPLCDDNQSLYLDDRTVARAPMEKTTKNTEFPESSFLNTLSH
NPPSLQSTMHVTNEIGDDFNNPHFSAESTTRWGGGATTATTTEEGVVKLSPILEATNEYEKS
MSTKYQSGLVSLIPSMHRTNVSTAATEKFKFGAIGLEDDITIPEQSRLVDSDVIDESQAIDY

DFKDLCDELMSTSLCKTTIDLMPDGIGDLSKPSLGLNIIPDPWNDQLLQGLIPSTLEGVIVA
SESKVFRKGSDVRIGNETYHLVKEIGKGAFAKAYKATMVSGDEVAVKVQSPAYKWEIHMLQE
VRRRLEAKGHDVCKDYMTIMTAAVFQNSSCVVTQYLPSGTLLDFLNTNKNNTVDRENIALQI
FHLVHSLHAIGVIHGDVKPDNILIANVSNRGPAPTLRLIDFGRAIDLSSLPPNTAFTDNCGT
SGFVCSQMKTNQPWNYHIDFNGVAGTLHVLLHSAYMKTMLNNKQEWVTTKKLPRWCDEKWSS
AFHDLLNFPTPTNDWCPSLQDSPLPHLIQLFDA

Bub3: XP_002129118.1 (Gene model: KH2012:KH.C5.394)
MANEFKLNNCPTDGISSVKFSPSTSQFLLASSWDMSVRLYDVTENSQRFKYEHKSPVLDCCF
SDSVHSWSGGLDGSVMMYDLNTGRETVVGRHNNSIRCVEYCSDTNVVVTGSWDQTIKLWDPR
SHNNIGSYSQPGKVFTMSVCGDHIIVGTCGKSVVVWDLRNMGYVEQRRESSLKYQTRCIKSF
PNKQGYVLSSIEGRVAVEYLDPSVEVQKKKYAFKCHRIKENGIEHIYSVHAIAFHQRYSTFA
TGGADGYVNMWDGFNKKRLCQFHLFPAAVSSLAFSNDGSMLAVASSPLYGAELSPSSNGEDA
IYIRHVTDAETKPKTSSGLA

Mps1: XP_002124942.1 (Gene model: KH2012:KH.L149.3)
MASQFTTEGLGDNPDKWFSMLQSCLNLQNGEEKNNLLKQIFVTANRSLTAEKHKTSEIYAKI
LAEEAAFVGSTNVNKGRNMFKHAVYVCRTIPIIHLTYAQFEVRNGHFDKALHILEFGKMVTG
CKLLFEQAISKLENGYVKFNSTLSMDLNQATPLKNITNTECNEKVTECQKRSYTDTALKTPP
SYRFRALDTKYYDIKTPSPPMLKMSPTNTFPNRFTFNMPTSISKSSVTNFTPTPASATKTTG
LTKGPQRVLRSLKSSLENADNDKEHDIGTHQTPYSLKHHPLVSNVEPQIPDSPKPPVIIDPS
PINPPTPIIPLKKEIFPYPSKQTPQPKLTPNPPKNLGTLNPEIRQNPSKSTPMKTDTSNLNH
NSMPPPKKFPPQRVPCSKPAEPQNGLHTWFNPNSAICVNNKHYLVIRELGEGGSSKVLQVFC
AETKAILALKKVSLKDCDESTKNEFTNEIEFLLKLRNNPHIVHLYDFELTPDFIHLVMECGS
TDLAKLLHSHKTQNSRLEVYEIIYFWKKMLLAVQTIHKHGVIHRDLKPANFLLVKGNLKLID
FGISNAINADATSVIKETQCGTLNYMAPEAILDMSGGYNPDSPKFKISPMADVWSLGCILYS
MLYGCTPFQHIKHQLLKLNAITNDQHRIEFPPFKDENFVKIVQKCLKRNPKHRPTVDQLLQF
S

Branchiostoma lanceolatum:

Mad1:
>lcl|BL00512_evm0
MMGDFNSYLAEGRRQKSNSSLLFDMGDTTAGKGDLTMQLDTTRDDSTVQWDRPETAKRNRVR
RLEQTQAIEKEGELLWTRSKVAKLETAITQLTTEKKRDKIEFEKGQETFRMQYERDLEQVND
LRHKLEFLVKEEKQAKEELAEERKKLTTIKTQMEKRIMVLQREKLCVQAELDELREASRSQI
SNLRNQVKRLEAELEMSDSELAEAQQQVQHHQGQAQARLEQVQQLQEDRIRAVTAEHKVKEL
ELQLNQVQESAAMAKLMSTRLSRYEQLEKENKKLLEQNKFYRETSENNQLLKEKLLSLETKY
GRAEHSNLELIKEVEDLRGRLKQWEGIDPSGGQIPRSPAEIAKWIAELQRKEVLLTEKTGQL
TSSARDSEAGQKAATGRLREVESQLFAATEKTNQQADLIKKLQRRLLLVTKDRDSIRNILVS
YESEVTRASSDDAMLQRLQDSEEAAQRYQRHMASLEEEMQRVNAQASEERIKAHKLEVELSQ
MKTQTPAPAPNTISSQEVTMLRKKVEDLEKDRQKLLEEKEILEARVEQRHLQGNYDPTKTKV
VHFGLNPTALRRQHRQEELEKLREECEKLRQRLRAAEEGGGADVTASILLTPSSKEMEDLKK
QLTTAETKNTRLKEVFQQKIHEFRQACYMLLGYKVDVVKDNNYKLMSMYAERQEDCLMFQMV
PGGELQLLETEFSNSQAVMMLVDLHLKTQHSIPAFLSALTLDLFSQQTIA

Mad2:
>lcl|BL13880_evm0
MAGVLQAAKNSITLKGSAEIVAEFFSYGINSILYQRGIYPPETFSRVQKYVTGHDRQRAEDV
LKQYWLLDKTVEKLVVVVTSIDTQEVLERWQFDIECDKTMTDDSKPREKSQKEINDEIKAVI

RQITASVTFLPLLEENCSFDLLIYTDKDLQVPETWEESGPHFIAKSEEVRLRSFSTSIHKVD
AMVCYKNAD

Bub1:

>lcl|BL16332_cuf4
MDPTENSWELSKENVQPLRQGRKIANLTAALQPVNGHDGGLTRVQHEKQAFEAELRMYSGDD
PFSVWDRYIKWTEQNYPKGGKDGSLSTLLERCLLFFKDDKRYSNDHRYLQAWIKFAGMTNQP
VDIYSFLHDQAIGAQVALFWEAWAWELEQEGDTKKADAVYMEGIQKQAQPVDLLVRKQKEFQ
ARVTRATLEGGVVQSADVSGTEAQRVTLGGLRGHGKKHQAPVNRTGAATKVYSSGLGVKPCA
PPQVGQAFSVFSEDNPQDQVIPTPTGEWTNPPLPRVTSRENTHKPGKWTEAKVSQKAVPAIP
FKAVSMNASSEFSIHVEEGAAQPQPTPQKPIEMGTQVLSARKPEKSLNPLRNMYMPPFEDEP
NKIPKYCKKFVYTGLEEFQFEELRAARHVEMEKKRKMEEEMRRQEEQRREMEKQHQLMLAQQ
RQQLQQMQQTFQQEQLALQQERKQMMEQCRQQMLRERAEMERLLVDRMRDTTLESAQLPGAA
PDGEPQDMPDSHAAQDSHIMPDSHAMQNSHVMQDSHAAQNSHTLKGSHVVQDSHIMLDSRQT
MQDSHIMQDSHAAQENDPSTAAGLTESHREHTVTKHLNFEESLASGLQPSVVSAMAEIEDYK
TDVEMVEPTPVKTAPQADTGDLRSGQFLLSSATPASTGKMVTSREQSLVKTPNTSTCSGPTP
STTPGRRPITDPSPTINTKAAMMELGEFFRDDIGFGAVGGSDGFEDMFAAGPSGGTASSGLS
FSAPQAQTTDFKGFTIFDESKDTGLEDRMDMKDNIVLEDQENNPPGGYQTGAKRRGLGVLQP
SQGIPVEEIKEVDDLDGIEPFADDNPDMTFASTGNQSSFCTMARMASTPFNRTGPELPELPA
SSIRPVHNSTRKALGMLEISTTEDLAEAGGSGAGRVSAESTEETFDMTVGNRPAAALSPIME
NSSEEKSQVSTDHGSTDSAPSLSVAMQSTSCADPSSESEAVATLNSPAEDEVFVTDVNPFDP
EVVSSLLAKVRGSLTSHQEYQEEDGHMPQIKPGVAINLGTELYHIDKLVGSGAFAKIYQASM
LDADDLTDLEGERKVTLKVQQPGCPWEFYINSQVHERVNRLQDPMDIKPSLMSIDAMHVFSD
GSVLVNEQHSCGSLLDIINLYRTAGKKMSEVMVVFYTLEILYIVEQLHRCHIIHADLKPDNF
LIRDGFEDKTECLFSGELRGLKLIDFGISLDMELFPPNTTFRARSNTSAFQCIEMQTGKPWS
YQADLYAIVSTVHCMLFGEYMKAYYEGGRWKITQTPRRTHDPVWRRFFDTFLNIPSCDELPS
LSYWISEFHQCWEEKKKTFDKEKWAQYTMYFEKH

Bub3:

>lcl|BL20329_cuf2
ICQSYNYCPTYLHATNMPTVAETPNEFKLNNCPTDGISAVKFSPTSSQLLLVSSWDCSVRLY
DVSGNNLRNKYTHSSAVLDCCFFDPVHPYSGGLDQMLKTCDLNTSQETVVGNHDAAIRCVEY
CPEVNVVITGSWDQTVKLWDPRTPCNAGSFSQPGKVYTMAVTGDRLIVGTAGRRVLVWDLRN
MGYVQQRRESSLKYQTRCIRAFPNKQGYVLSSIEGRVAVEYLDPSPEIQKKKYAFKCHRIKE
DGIEKIYPVNAIAFHSLHNTFATGGSDGFVNIWDGFHKKRLCQFHRYPTSIASLAFSHDGSV
LAIASSYMYEELDVEHPEDAIYIRNVTDQETKPK

Mps1:

>lcl|BL08039_evm0
MVEKSDRQIMSRLKSLQSRLQSIYSRDEKDDTNNTGNLTGTGTYNLTDHTSWIDRISAAGNK
PSDWLEFLTQAQGRTSFSSPQARYTYLLTVYDKALHHMPVELCRRDESYAKILIQLAHIKGD
HNEEDGQTGFKFARVNAKTFAFVHVAAAQFELSRGREEKAQSILQKGLDVGAEPTEMLNTAL
IRLGNKEKLLMEDKENVWGICYPDSVQPSFSAAKPKGPALTKQQPPQNQTTVDPSPSLVSMD
STIVFQKQGGCRNDSGGSSSLDENDTLPLLWGHREKPPQLSRSTWSATKLPGRELHTPQSQT
QRVVTSHTEGPNANKRQPARVPLRVKRSSLPKLREMSTEDVNDDDSDDTDCFLGVKPLKNIS
PPTEGMDTLAARAKPPLPFTGRKVSPESNTNTTNDSKAFKGLSLCKFSKSSSLLSHTTPRGQ
MLSKETSPTENTASSKPTEPLRETSKAPLVSNPGHHSTHKNPITDSARKAQSISPTSTVVSF
PERQPPCAKVPSASDTADAATVAFNSMQVPGAQASHMHIPPVSSNLPPGNKTIEHLPSQHAH
QAPKMLDSLKSVVPEVRSNSLHTVGISHEVSGGQGSVQLGTRSGPATGPTVVPKNSQMPTPN
IVNQNTMPGFFTPSASLPGAQQHVQQPHSQHFQQVAPPPQPQFATPRMPCAPTERSDVICVK
GKPFRILSIIGKGGSSKVYQVYDEKGKVYALKLVNLEEGDETTVQGYINEITHLSNLQHSPR
VIKLYDFEVTDERIVLVMEKGSVDLATFLRNKKKQQGSIADDVLWFYWRHMLEAVDTIHRQG

IVHSDLKPANFLFVDGDLKLIDFGIANAIQSDKTSVIKDQQVGTLNYMSPETIREYNPAQYR
DGNSKKLFKINCRSDVWSLGCILYYMVYGKTPFQHIPNHFAKLQAIVDPNYEIQFPPIKNHL
LLDMLKKCLIRDPYNRPSTADLLAHPYLNSERSSGAKEQPCPAAPALPMTADQIQQLLTQLS
QAQSMSPNSISTVSKGLAAQLQSGQAPDLSAVLKKRQGPSQDKENLPPQHLLRKGPGCTLQH
ALPRPPSEHTLGRP
