# Supplementary material for: The Spindle Assembly Checkpoint Functions during Early Development in Non-Chordate Embryos
Source: Cells. 2020 Apr 28;9(5):1087. doi: 10.3390/cells9051087 (PMC7290841; doi:10.3390/cells9051087)
Supplement: Supplementary file 1 [file cells-09-01087-s001.zip › ChenevertSupplementary/Chenevert_TableS3.pdf]

Table S3: Value associated with Figure 2

Paracentrotus lividus			
	mitosis (minutes)		
embryo	DMSO	nocodazole	noco+ rev
1	20	100	14
2	24	100	22
3	22	100	21
4	19	100	16
5	22	100	29
6	22	100	15
7	20	60	25
8	20	92	28
9	20	93	18
10	12	94	15
11	26	93	28
12	18	106	26
13	22	100	26
14	24	104	26
15		90	24
16		86	26
17		92	22
18		90	24
19		92	
20		94	
21		114	
22		94	
23		110	
24		92	
25		98	

Clytia hemisphaerica			
	mitosis (minutes)		
embryo	DMSO	nocodazole	noco+ rev
1	11	45	28
2	11	52	26
3	11	52	14
4	11	67	19
5	12	31	31
6	11	30	15
7	9	35	19
8	13	51	18
9	14	40	24
10	12	38	24
