# Supplementary material for: The Spindle Assembly Checkpoint Functions during Early Development in Non-Chordate Embryos
Source: Cells. 2020 Apr 28;9(5):1087. doi: 10.3390/cells9051087 (PMC7290841; doi:10.3390/cells9051087)
Supplement: Supplementary file 1 [file cells-09-01087-s001.zip › ChenevertSupplementary/Chenevert_TableS1.pdf]

Table S1:Morphometric data for analysed embryos

Egg diameter (µm)					
Ch	Pl	Pm	Ha	Mg	Bl
224	95	136	154	64	133
210	95	131	153	65	133
218	91	133	163	65	136
200	93	132	162	67	130
196	91	131	160	64	134
200	95	128	158	64	136
190	90	132	159	64	133
216	91	129	158	66	133
208	92	131	157	64	136
200	91	129	160	64	134
216	91	132	160	64	133
210	90	128	156	62	135
218	93	127	154	64	133
234	89	134	157	62	133
214	94	128	165	62	135
212	91	132	162	65	135
202	90	133	161	64	135
200	92	129	161	65	136
212	89	127	161	64	134
235	93	128	156	65	135
218	91	130	160	66	132
218	91	132	160	65	132
198	90	129	162	65	133
220	92	131	157	64	134
202	92	133	164	66	133
210	95	128	160	65	133
216	90	129	165	64	133
208	89	131	150	65	133
202	91	131	155	67	132
207	90	130	149	65	134
218	91	128	149		
232		132	149	64,5	133
	91,5483871		154	1,224275531	1,417866294
211	1,804415135	130,4375	152		
10,95665949		2,19878999	147		
			146		
			152		
			152		
			150		
			150		
			150		
			160		
			143		
			154		
			157		
			154		
			154		
			149		
			152		
			153		
			150		
			151		
			148		
			148		
			146		
			155,0727273		
			5,493614291		

avarage	standard dev

Nuclear diameter (µm)				
Ch	Pl	Pm	Mg (Nup)	Mg (BF)
10	17,5	16	17	15
10	15,5	16	17	15
8	15,5	16	17	16
7	15	18	18	16
8	14,5	16	15	15
9	15	12	19	15
8,5	13,5	12	18	15
8	12,5	14	14	16
9	15	16	15	18
8,5	15	16	17	16
9	15	16	17	16
7,6	15	12	16	16
8	13,5	10	18	17
10	13,5	12	18	16
7	14	12	19	18
	16,5	14	17	18
8,5	13	14	17	15
0,993167132	15	10	17	16
	15	10	17	19
	14,5	14	17	17
	15	12	18	16
	15	15	20	17
	13,5	15	18	16
	14	12	19	14
	13	14	17	15
	16	14	19	15
	14,5	12	17	16
	13,5		17	15
	14,5	13,7037037	19	17
	12,5	2,180592982	19	
	14		16	16,06896552
	14		19	1,162848549
	11		20	
			22	
	14,37878788		19	
	1,25018938		15	
			17,5	
			15	
			12,5	
			12,5	
			17	
			15,5	
			18	
			15,5	
			18	
			15,5	
			15,5	
			14	
			17,5	
			14	
			13,5	
			12	
			13,5	
			12	
			11	
			13,5	
			16,5625	
			2,296365706	

Volume blastomere at 2-cell stage (cell Mask Orange)					
Ch x10^5	Pm 10^5	Pl 10^5	Bl 10^5	Mg (Large) 10^4	Mg (small) 10^4
18,4	6,1	2,3	5,2	8,3	4,5
20	7,5	2,1	5,18	7,9	4,6
19,2	7,2	2,3	5,2	8,2	4,5
16,3	7,3	2,1	5,12	8,4	4,3
17,2	7,3	2,4	5,23	8,2	4,475
19	7,5	2	5,2	8,25	4,5
16,3	7,6	2,2	5	0,21602469	0,125830574
16	7,2	2,3	4,5		
24	7,1	2,05	4,7		
22	7,2	1,8	5		
22	7,2	1,9	5,5		
20	6,9	1,7	4,8		
17,4	6,3	1,7	4,8		
21,5	6,1	1,8	5,7		
18,5	6,7	1,6	5,3		
20	6	1,7	4,5		
20	5,8	1,8	4,6		
20,4	5,8	1,8	4,6		
23,8	5,5	1,9	5,007222222		
24	5,4	1,8	5,06		
18,6	5,7	1,9625	0,346304958		
19,1	5,5	1,9			
16,2	5,7	0,242180337			
19,56086957	5,9				
19,2	6,520833333				
2,468481154	6,5				
	0,764414619				

Volume M. galloprovincialis blastomere at 2-cell stage (mathematical)	
Large Blastomerex 10^4	Small Blastomere x 10^4
12	3,5
10,5	4,2
11,4	4,7
10,5	3,9
11,6	3,6
10,8	3,9
10,1	4,2
9,3	5
11,2	3,8
9,9	4,7
9,6	3,5
9,7	4,9
11,3	4,6
10,7	4,4
10,7	3,7
13,8	4,5
9,7	3,6
11,9	4
9,9	4,2
11	4,1
11,7	4,5
11,8	3,2
11	4
11,3	4,4
11,5	4,4
10	4,5
11,4	4,4
11	4,7
10,90357143	4,182142857
11	4,2
0,95935246	0,465119317
