# Supplementary material for: The Spindle Assembly Checkpoint Functions during Early Development in Non-Chordate Embryos
Source: Cells. 2020 Apr 28;9(5):1087. doi: 10.3390/cells9051087 (PMC7290841; doi:10.3390/cells9051087)
Supplement: Supplementary file 1 [file cells-09-01087-s001.zip › ChenevertSupplementary/Chenevert_TableS4.pdf]

Table S4: Values associated with Figure 3

*P. mammillata*_time (minutes)												
DMSO				Nocodazole								
embryo	cell	M	I	embryo	cell	M 1	I1	M 2	I 2	M 3	I 3	M 4
1	1	13	16	1	1		16	14	20	12	30	16
1	2	14	15	1	2		16	14	20	12	28	16
2	1	12	16	2	1	22	26	22	30	28	50	28
2	2	12	16	2	2	22	26	20	32	24	48	
3	1	13		3	1	14	22	18	24	18	42	12
4	1	15		3	2	16	20	18	26	14	38	12
4	2	16		4	1	20	18	20	18	14	36	8
5	1	13		4	2	16	20	20	20	14	36	14
5	2	18		5	1		16	12	24	12	36	14
6	1	12		5	2		16	14	22	12	34	16
6	2	12		6	1		14	16	26	10	44	12
7	1	13		6	2		16	16	24	10	44	12
7	2	13		7	1		20	14	22	10	32	14
8	1	13		7	2		20	14	22	10	34	18
8	2	14		8	1	16	22	12	24	12	36	12
9	1	8	16	8	2	14	22	14	20	12	30	14
9	2	10		9	1	14	20	12	26	10	36	12
10	1	8	14	9	2	12	20	12	30	10	40	12
10	2	8		10	1	21	14	20	22	17	34	16
11	1	10	16	10	2	20	16	17	24	17	35	14
11	2	10		11	1	21	14	18	22	16	35	13
12	1	12		11	2	20	15	16	25	14	33	16
12	2	12		12	1	17						
13	1	10		13	1	19	18	17	25	14	39	14
13	2	12		13	2	19	18	17	26	17	36	15
14	1	12	16	14	1	19	19	18	24	18	35	16
14	2	10		14	2	20	18	21	21	16	38	
15	1	12	16	15	1	18	18	18	23	14	37	13
15	2	12		15	2	20	17	17	24	16	36	16
16	1	10	10	16	1	22	24	16	20	12	26	12
16	2	10		16	2	14	32	14	22	12	30	10
17	1	8	8	17	1	12	6	12	12	14	20	12
17	2	8		17	2					14	22	12
18	1	8	8	18	1	14	8	12	12	14	20	12
18	2	8		18	2					12	18	12
19	1	10	10	19	1	12	10	10	14	12	20	10
19	2	12		19	2	12	10	10	12	12	24	10
20	1	8	8	20	1	12	10	12	12	12	24	12
20	2	8		20	2	12	10	12	12	12	24	10
21	1	12	8	21	1	12	10	12	14	12	24	12
21	2	12		21	2	10	12	12	14	12	24	14

22	1	8	
22	2	8	
23	1	10	
23	2	12	
24	1	14	19
25	1	13	19
26	1	13	18
27	1	16	16
28	1	16	16
29	1		14
30	1	12	14
31	1		13
32	1		15
33	1	14	14
34	1	11	16
35	1		14
36	1	11	15
37	1		14
38	1		16
39	1	13	16
40	1	12	16
41	1	13	10
42	1	13	10
43	1	13	10
44	1	13	10

22	1		14	20	20			
22	2		14	22	16			
23	1			18	30	18	42	16
23	2			18	32	16	44	16
24	1	20	18	18	26	16	42	16
24	2		18	18	26	18	40	18
25	1	18	22	18	26	16	38	
25	2	20	20	16	28	18	42	
26	1	20	20	18	28	14	42	14
26	2	20	20	18	26	16	40	16
27	1	20	22	16	26	14	40	14
27	2	18	22	18	26	16	42	16
28	1	22	18	22	20	20	34	20
28	2	22	16	22	22	20	32	20
29	1	24	16	20	22	16	40	16
29	2	22	18	16	26	16	34	16
30	1	18	18	16	28	14	38	14
30	2	18	20	16	26	14	38	14
31	1	20	20	18	24	14	36	14
31	2	20	20	18	24	12	38	12
32	1		16	18				
32	2		16	18				
33	1		18	16				
33	2		16	20				
34	1		16	18				
34	2		16	18				
35	1			20	22	20	30	20
35	2			20	22	22	36	22
36	1	20	22	18	36	18	42	18
36	2	20	22	22	28	16	40	16
37	1	14	28	14	40	20	48	20
37	2	14	28	14	42	20	46	20
38	1	20	17		26	18	36	20
39	1	19	20		29	19	40	17
40	1	19	20		29	20	35	18
41	1	18	20		29	17	39	15
42	1	16	18	16	28	12	40	16
42	2	16	18	16	28	12	38	14
43	1	18	18	16	30	14	34	16
43	2	18	12	18	28	16	36	14
44	1	20	16	16	28	16	38	14
44	2	18	16	18	24	16	38	14
45	1			20	20	14	34	16
45	2		12	18	20	14	32	16
